# The NmpRSTU multi-component signaling system of *Myxococcus xanthus* regulates expression of an oxygen utilization regulon

**DOI:** 10.1128/jb.00280-24

**Published:** 2025-01-27

**Authors:** Colin T. McAllister, Allison M. Ronk, Mason J. Stenzel, John R. Kirby, Daniel J. Bretl

**Affiliations:** 1 Department of Microbiology, University of Wisconsin-La Crosse 14750 , La Crosse, Wisconsin, USA; 2 Department of Microbiology and Immunology, Medical College of Wisconsin 5506 , Milwaukee, Wisconsin, USA; University of Massachusetts Chan Medical School, Worcester, Massachusetts, USA

**Keywords:** *Myxococcus*, transcriptional regulation, NtrC, two-component signaling systems

## Abstract

*Myxococcus xanthus* has numerous two-component signaling systems (TCSs), many of which regulate the complex social behaviors of this soil bacterium. A subset of TCSs consists of NtrC-like response regulators (RRs) and their cognate histidine sensor kinases (SKs). We have previously demonstrated that a multi-component, phosphorelay TCS named NmpRSTU plays a role in *M. xanthus* social motility. NmpRSTU was discovered through a screen that identified mutations in *nmp* genes that restored Type-IV pili-dependent motility to a nonmotile strain. The Nmp pathway begins with the SK NmpU, which is predicted to be active in the presence of oxygen. NmpU phosphorylates another SK, NmpS, a hybrid kinase containing an RR domain and a HisKA-CA domain. These two kinases work in a reciprocal fashion: when NmpU is active, NmpS is inactive, and vice versa. Finally, the phosphorelay culminates in NmpS phosphorylating the NtrC-like RR NmpR. To better understand the role of NmpRSTU in *M. xanthus* physiology, we determined the NmpR regulon by combining *in silico* predictions of the NmpR consensus binding sequence with *in vitro* electromobility shift assays (EMSAs) and *in vivo* transcriptional reporters. We identified several NmpR-dependent, upregulated genes likely to be important in oxygen utilization. Additionally, we demonstrate NmpRSTU plays a role in fruiting body development, suggesting a role for oxygen sensing in this behavior. We propose that NmpRSTU senses oxygen-limiting conditions, and NmpR upregulates genes associated with optimal utilization of that oxygen. This may be necessary for *M. xanthus* physiology and behaviors in the highly dynamic soil where oxygen concentrations vary dramatically.

**IMPORTANCE:**

Bacteria use two-component signaling systems (TCSs) to respond to a multitude of environmental signals and subsequently regulate complex cellular physiology and behaviors. *Myxococcus xanthus* is a ubiquitous soil bacterium that encodes numerous two-component systems to respond to the conditions of its soil environment and coordinate multicellular behaviors such as coordinated motility, microbial predation, fruiting body development, and sporulation. To better understand how this bacterium uses a two-component system that has been linked to the sensing of oxygen concentrations, NmpRSTU, we determined the gene regulatory network of this system. We identified several genes regulated by NmpR that are likely important in oxygen utilization and for the *M. xanthus* response to varied oxygen concentrations in the dynamic soil environment.

## INTRODUCTION

*Myxococcus xanthus* is a ubiquitous soil bacterium that displays complex coordinated behaviors that include two distinct forms of motility, mechanisms of kin selection, and microbial predation (1–5). During periods of low nutrient concentrations, *M. xanthus* forms multicellular fruiting bodies containing spores. As a model for multicellular behaviors, *M. xanthus* has been widely studied with the aim of understanding the underlying molecular mechanisms that control this development within single cells and between cells in the developing population (6). Because development is integral to the life cycle of *M. xanthus,* it is influenced by a wide-ranging set of genes under strong evolutionary pressures and include a wide repertoire of signal transduction systems (6, 7). While development is well understood to be triggered by a depletion of nutrients, less is known about other environmental and cellular signals, ligands, and/or other molecular cues that influence development and related behaviors. This lack of understanding of the specific signals is particularly striking given the substantial cellular signaling networks of *M. xanthus*. For example, *M. xanthus* encodes numerous two-component signaling systems (TCSs), including ~135 histidine sensor kinases (SKs) and ~130 response regulators (RRs), which represent a disproportionally large number of signaling genes relative to other bacteria, even given its large genome size (~9.3 Mb) (8–11).

Regulation of development by *M. xanthus* requires a network of cellularly derived signals (12), which are directly produced and sensed by the population of *M. xanthus* cells. For example, cell-density-dependent production of secreted amino acids is necessary for the earliest steps in development (13–16). These secreted amino acids have been proposed to act as a form of quorum sensing and are thought to be directly sensed by a TCS known as SasSR (17, 18). Another cellularly derived signal, which consists of a membrane localized protein, CsgA, then initiates a signaling cascade that includes activation of two key RRs, MrpB (19, 20) and ActB (21). Notably, SasR, MrpB, and ActB are all members of a family of RRs known as the NtrC-like activators (Nla) due to their homology to NtrC of *Escherichia coli*. Several other NtrC-like RRs have critical roles in *M. xanthus* development including Nla4, Nla6, Nla18, and Nla28 (22–27). Yet other NtrC-like RRs play a role in the regulation of *M. xanthus* motility, a key social behavior necessary for development. Regulation of motility includes the expression of Type-IV pili (T4P) via the evolutionarily conserved TCS PilSR (28, 29) and the regulation of the assembly of T4P via an unknown mechanism controlled by the TCS PilS2R2 (29).

**Fig 1:**
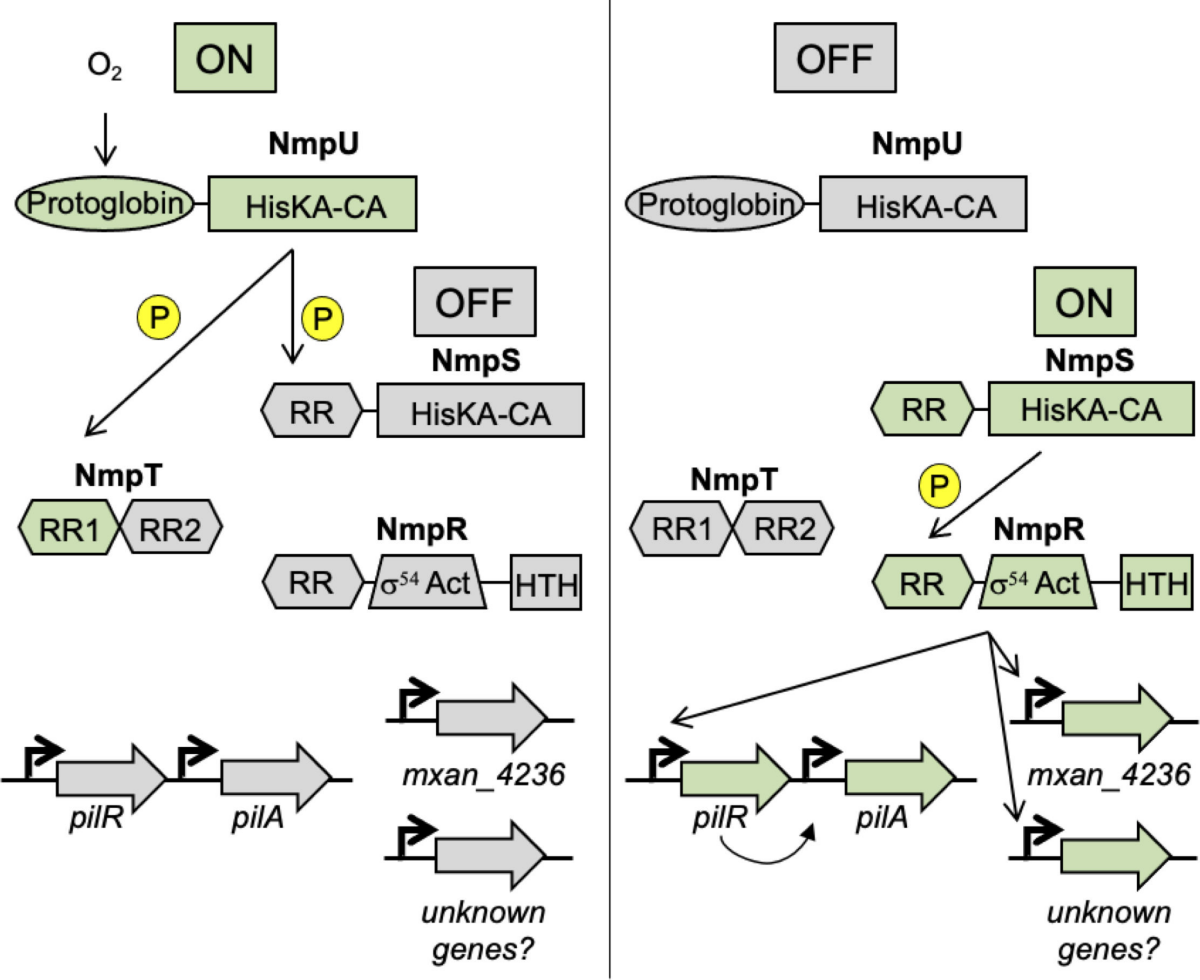
Model of the NmpRSTU multi-component signaling pathway. The presence of oxygen is sensed by the SK NmpU. During high oxygen conditions, NmpU phosphorylates NmpT, a likely phosphosink protein. NmpU also phosphorylates the hybrid SK, NmpS. When NmpS is phosphorylated, the remaining portion of the pathway is “OFF.” When oxygen is limiting, NmpU switches to an “OFF” state, NmpS is no longer phosphorylated, and NmpS phosphorylates NmpR. We have previously shown that NmpR binds to the promoter region of *pilR* and *mxan_4236*, and in this study identified previously unknown genes regulated by NmpR. The depiction of this model is modified from reference (30).

Our lab has previously demonstrated that yet another NtrC-like RR plays a role in the social behaviors of *M. xanthus*. This TCS was discovered serendipitously while investigating the role of PilS and PilR in the regulation of T4P-dependent social motility (30). The *M. xanthus* Δ*pilR* strain is nonmotile due to its inability to express *pilA*, encoding the PilA monomer that polymerizes to form T4P. However, upon extended incubation of this strain, we observed suppressor mutants with restored motility. The mutations in 12 independent suppressor strains mapped to a single genetic locus encoding a previously uncharacterized TCS that we named the Nmp (NtrC Modulator of Pili) system (30). The Nmp system (Fig. 1) is a multi-component system encoded by four genes: *nmpR*, *nmpS*, *nmpT*, and *nmpU* (*mxan_4240* [MXAN_RS20585], *mxan_4244* [MXAN_RS20605], *mxan_4245* [MXAN_RS20610], and *mxan_4246* [MXAN_RS20615], respectively). NmpU is a cytoplasmic, soluble SK and the initial SK in the pathway that determines the overall “ON/OFF” state of the system. The NmpU homolog in the related bacterium *Anaeromyxobacter* sp. Fw109-5 responds to oxygen concentrations via its oxygen-binding protoglobin sensing domain (30–35). When active, NmpU autophosphorylates and subsequently passes that phosphoryl group to the next proteins in the pathway, NmpT and NmpS (30, 35). Little is known about NmpT, but it may be a phospho-sink protein that modulates the overall flow of phosphoryl groups through the system, because it consists solely of two RR receiver domains. The next protein in the pathway is NmpS, an atypical hybrid SK with a structure consisting of an N-terminal RR receiver domain and a C-terminal HisKA-CA domain. Based on biochemical and genetic evidence, we proposed a model (Fig. 1) wherein NmpU phosphorylates NmpS on its N-terminal receiver domain and prevents the autokinase activity of NmpS, consistent with other hybrid SKs with similar domain architecture (30, 36, 37). Therefore, when NmpU is “ON,” NmpS is “OFF,” and vice versa. When oxygen concentrations are depleted, NmpU switches to an “OFF” state, the receiver domain of NmpS is unphosphorylated, and NmpS autophosphorylates at the conserved histidine residue on its HisKA-CA domain. Finally, NmpS transfers the phosphoryl group to the output of the system, the NtrC-like RR NmpR. Collectively, we have proposed that the Nmp system senses oxygen concentrations and that as oxygen concentrations are depleted, NmpR is ultimately phosphorylated to regulate genes to respond to this stress. It is important to note that *M. xanthus* is generally considered to be an obligate aerobic organism; thus, under almost all laboratory conditions, *M. xanthus* is grown and phenotypically assayed under oxygen replete conditions (i.e., NmpR “OFF”). All the mutations we characterized in our previous study are predicted to turn NmpR “ON,” even in the presence of oxygen.

Following the discovery of the NmpRSTU system and our biochemical and genetic characterization of this TCS (30), we sought to determine the NmpR-dependent regulon. We have previously shown that NmpR binds to a promoter upstream of *pilR* (*mxan_5748* [MXAN_RS28040])*,* as well as the promoter of the first gene of its own putative operon, *mxan_4236* [MXAN_RS20570] (30). Using these known binding sequences, we combined *in silico* predictions of the NmpR consensus binding sequence with electromobility shift assays (EMSAs). We show that genes identified by this strategy are indeed upregulated in an NmpR-dependent manner and that several of the identified genes are predicted to be important in oxygen utilization. These results are consistent with the evidence suggesting that NmpU senses oxygen concentrations. Finally, we demonstrated that NmpRSTU signaling plays a role in fruiting body development. Overall, we propose that the *M. xanthus* NmpRSTU pathway senses oxygen depletion and upregulates genes associated with optimal utilization of oxygen under limiting conditions. This is likely necessary for *M. xanthus* physiology and cooperative behavior in the soil environment where oxygen concentrations vary dramatically (38).

## MATERIALS AND METHODS

### Bacterial strains and plasmid construction

All *M. xanthus* strains used in this study are listed in Table S1. *E. coli* DH5α and *E. coli* BL21(DE3) strains were used for cloning and protein production, respectively, and were routinely grown at 37 °C in lysogeny broth (LB) (Becton-Dickinson, Franklin Lakes, NJ, USA). For all *M. xanthus* experiments, the *M. xanthus* DZ2 strain was used as the wild-type (WT) strain (39, 40) and was routinely cultivated in Casitone Yeast Extract (CYE) medium (41) at 32 °C with shaking at 220 RPM or on CYE agar plates at 1.5% agar, except in motility assays where 0.5% agar was used. Kanamycin was used at a final concentration of 50 µg/mL, tetracycline at 10 µg/mL, or spectinomycin at 500 µg/mL, when required for *M. xanthus* selection. All plasmids were constructed by standard laboratory cloning methods. Plasmids and primers used in this study are listed in Table S2. For cloning, *M. xanthus* DNA was purified by phase extraction. Briefly, cell pellets were suspended in buffer containing 75 mM NaCl, 25 mM EDTA, and 10 mM Tris, pH 7.5. Cells were lysed with 1% sodium dodecyl sulfate, 250 µg/mL proteinase K (New England Biolabs, Ipswich, MA, USA), and RNase A (New England Biolabs, Ipswich, MA, USA). DNA was extracted with chloroform and precipitated with isopropanol. PCR reactions were performed using Failsafe polymerase with Buffer K (Epicentre Technologies, Madison, WI, USA). All primers were supplied by Integrated DNA Technologies (Coralville, IA, USA) or Eurofins Genomics (Louisville, KY, USA). PCR products were directly cloned into plasmids and then verified by Sanger Sequencing at Eurofins Genomics (Louisville, KY, USA). Final plasmids were electroporated into the appropriate *M. xanthus* strains and confirmed with selection and PCR. When necessary, mutations to plasmids were carried out with the Q5 Site-Directed Mutagenesis Kit (New England Biolabs, Ipswich, MA, USA) per the manufacturer’s instructions.

### *In silico* prediction of NmpR-binding sites

**Fig 2:**
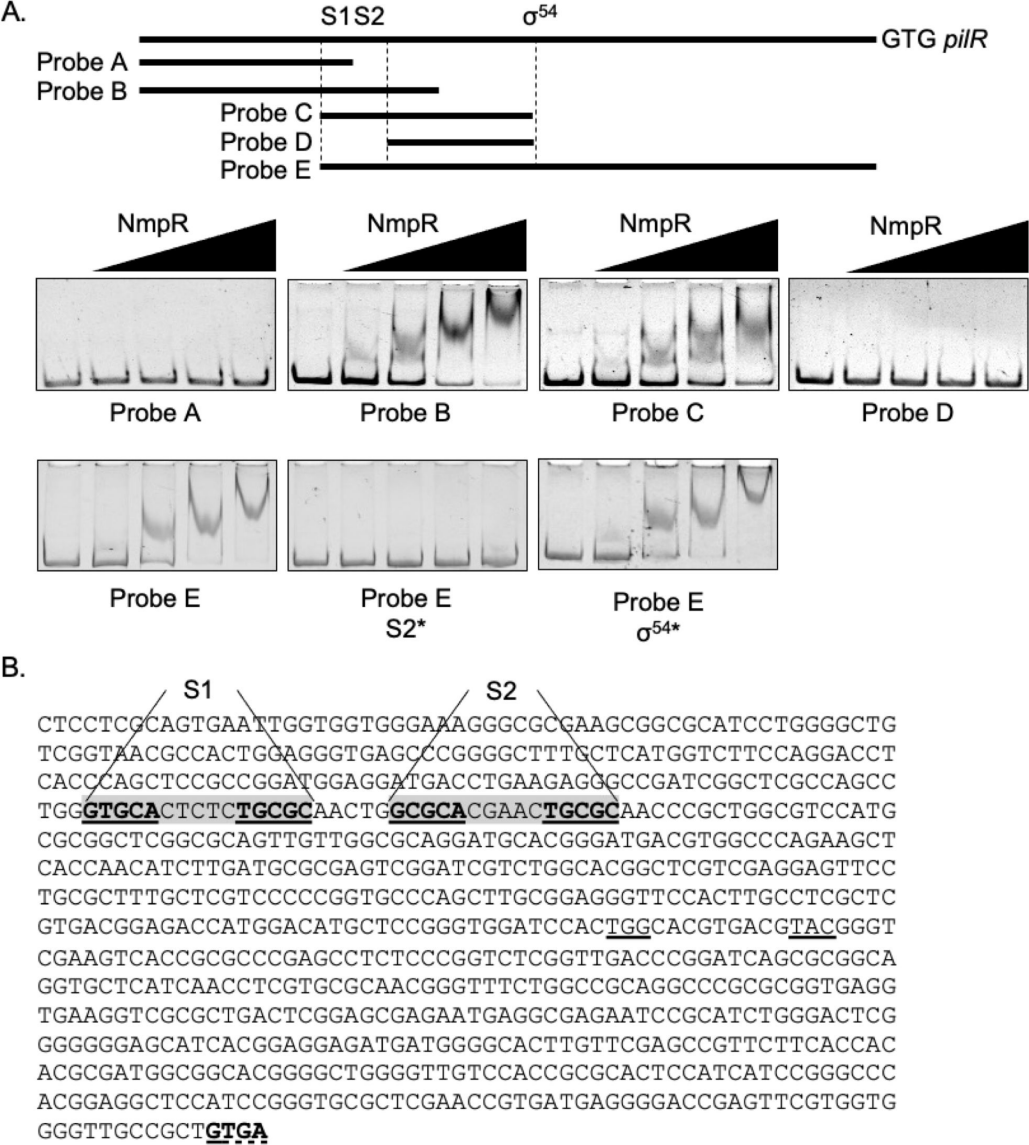
NmpR binds a consensus sequence of GCGCA-N_5_-TGCGC in the *pilR* (*mxan_5784*) promoter. (**A**) DNA probes containing different lengths and locations upstream from the *pilR* start codon were generated by PCR and used in electromobility shift assays with increasing amounts of NmpR (2, 8, 16, or 32 pmol per reaction, left to right as indicated). NmpR binds and shifts DNA probes that contain S1 and S2 in a reproducible, concentration-dependent, step-wise fashion. The S2* probe had the GCGC portion of the repeat changed to all adenines. The σ^54^* probe had the predicted σ^54^-binding site (underlined in [**B**]) changed to all adenines. (**B**) The entire sequence included in Probes A–E is shown with the NmpR-binding site shaded gray, and the consensus sequence, bold and underlined. The start codon of *pilR* overlaps with the stop codon of *pilS*, shown in bold and underlined (dashed and solid, respectively).

**Fig 3:**
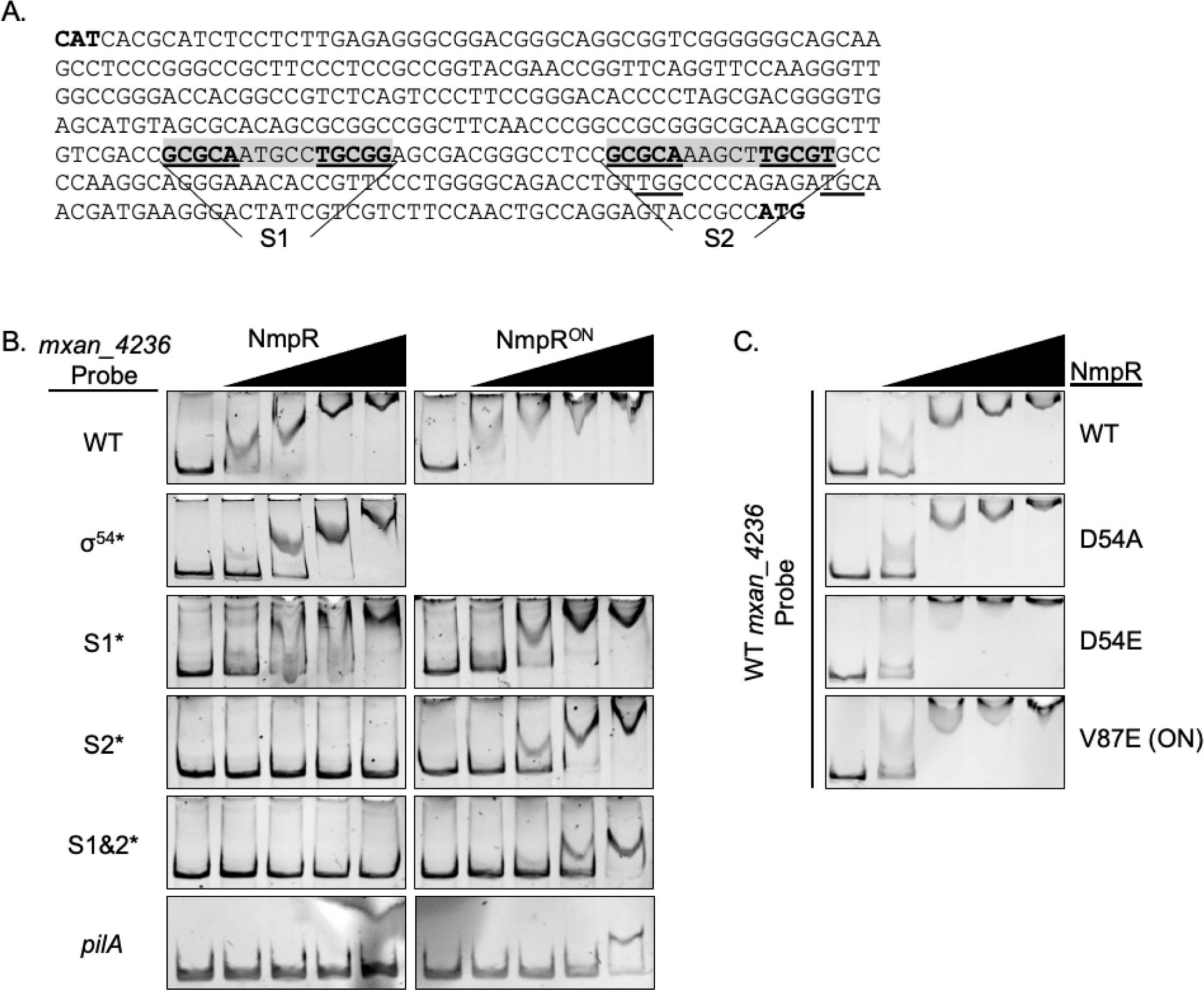
NmpR binds to the consensus sequence in the *mxan_4236* promoter. (**A**) The intergenic region upstream of *mxan_4236* is shown with S1 and S2 as indicated. The σ^54^-binding site is underlined, and the divergent start codons of *mxan_4235* and *mxan_4236* are in bold, CAT and ATG, respectively. (**B**) NmpR shifts the *mxan_4236* upstream region (585 bp) in the same pattern as the *pilR* promoter (Fig. 2). When S1 was mutated to all adenines (S1*), a reproducible smear of an NmpR shift was observed. Mutation of S2 to all adenines (S2*) abolished the binding of the wild-type NmpR. In contrast, the NmpR^ON^ variant maintained the step-wise shift of binding when either S1 or S2 was mutated. The σ^54^* probe had the predicted σ^54^-binding site (underlined in [**A**]) changed to all adenines. The *pilA* promoter was used as a negative control. (**C**) The phosphomimetic NmpR^D54E^ and NmpR^ON^ (V87E) variants shift the *mxan_4236* wild-type probe with higher-order oligomers compared to the wild-type NmpR or the NmpRD54A (non-phosphorylatable) variant.

The NmpR-binding region upstream of the σ^54^-binding site of *mxan_5784* (Fig. 2) and the intergenic region upstream of *mxan_4236* (Fig. 3) (30) were used as inputs for FAIR: Finding All Identical Repeats (42). The outputs of the search were manually curated to identify sequences characteristic of NtrC-like DNA binding as outlined in the results. Following identification of the GCGCA-N_5_-TGCGC consensus, this sequence was then used to search the *M. xanthus* genome using Pattern Locator (43). The *M. xanthus* DK1622 strain was used for this analysis as that was the genome available via Pattern Locator and due to its well-annotated genome (11). Initial input for the Pattern Locator included the consensus sequence allowing for two errors. The output was manually curated as outlined in the results. As further NmpR-binding sequences were identified, the Pattern Locator search was refined by using the motif search option. To predict potential σ^54^-binding sites, we also used Pattern Locator using the σ^54^-consensus sequence (44).

### Protein purification

All proteins were purified based on a standard affinity chromatography protocol (45). Briefly, plasmid constructs conferring an N-terminal 6×His-tag (pET-28a[+] backbone [Novagen]) were transformed into *E. coli* BL21(DE3) (Life Technologies, Grand Island, NY, USA). The transformants were grown in Terrific Broth to log phase at 37°C at which time they were induced for protein production with 1 mM isopropyl-D-1-thiogalactopyranoside (IPTG) overnight at 16°C. The cells were pelleted at 6,000 × *g* at 4°C for 20 min. The pellet was either processed immediately or stored at −20°C. The pellet was suspended in 10 mL of cell lysis buffer (25 mM Tris, pH 7.6, 125 mM NaCl, 5 mM imidazole, 0.1% Triton X-100, and 0.625 g of CelLytic Express [Sigma-Aldrich, St. Louis, MO, USA]) and was rocked for at least 1 h and then pelleted at 8,000 × *g* for 30 min. One milliliter of HIS-Select Cobalt Affinity Gel (Sigma-Aldrich, St. Louis, MO, USA) was equilibrated with deionized H_2_O and then with wash buffer (25 mM Tris, pH 7.6, 125 mM NaCl, and 1 mM imidazole). Clarified cell lysates were added to the affinity columns and allowed to flow through under gravity, washed with 20 mL buffer containing 10 mM imidazole, with 20 mL buffer containing 20 mM imidazole, and then eluted with 5 mL of buffer containing 250 mM imidazole. Eluted proteins were dialyzed in 25 mM Tris, pH 7.6, 125 mM NaCl, 1 mM dithiothreitol, 0.1% Triton X-100, 50% glycerol, and 0.5 mM EDTA. Protein purity was assessed by standard denaturing gel electrophoresis, and concentration was determined using the Bradford reagent. All purified proteins were used immediately or stored at −20°C.

### Electromobility shift assays

To demonstrate NmpR binding to specific DNA sequences, EMSAs were performed essentially identically to that previously published (30). DNA probes as indicated were generated by PCR. PCR products were separated on a 1% agarose gel to confirm fragments of the correct size, followed by purification using a PCR DNA Fragments Extraction Kit (IBI Scientific, Dubuque, IA, USA) and quantified by spectrophotometry. Probes were diluted in 10 mM Tris-HCl, pH 8.5, to 100 fmol/µL with a final quantity of 200 fmol used per reaction. Probes were incubated with an increasing amount of NmpR (0, 2, 4, 8, and 16 pmol) in a reaction containing a final concentration of 20 mM HEPES, pH 7.6, 1 mM EDTA, 10 mM (NH_4_)_2_SO_4_, 1 mM dithiothreitol (DTT), Tween 20 0.2%, and 30 mM KCl. NmpR was incubated with the DNA probes for 15 min at room temperature and then loaded on 8% acrylamide nondenaturing PAGE gels (8% acrylamide, 10 mM Tris-HCl, 1 mM EDTA, 400 mM glycine). Gels were resolved at 60 v for ~1.5 h, then stained with ethidium bromide (1 µg/mL in H_2_O) for 15 min prior to visualization. To generate mutated probes, plasmids containing the corresponding promoter (see below) were mutated with the Q5 Site-Directed Mutagenesis Kit, sequence verified, and then those plasmids were used as PCR templates to generate the EMSA DNA probes. For each assay, three replicates were conducted, and the presented images are representative of reproducible independent reactions.

### β-galactosidase assays

To assess the NmpR-dependent transcriptional activity *in vivo*, *lacZ* reporters were constructed as previously described (30). Each promoter was amplified by PCR and cloned into pCD127, a pSUM117 derivative containing a promoterless *lacZ* and an *attP*Mx9 attachment site for integration (30). Resulting plasmids were transformed into wild-type *M. xanthus* or mutant derivatives, including Δ*nmpR*, Δ*pilR*, or Δ*pilR* NmpRV87E (NmpR^ON^). In some assays, the *mxan_4236* promoter was mutated using the Q5 Site-Directed Mutagenesis Kit to change the GCGC repeat of each half site of Site 1 and/or Site 2 to all adenines, conserving the promoter spacing but abolishing the NmpR-binding sequence. To quantify the β-galactosidase activity in this study, all strains were grown to log phase growth (OD_600_ of ~0.8–1.0), cells were pelleted, suspended in Z-buffer, and lysed by bead beating with ~100 µL zirconium beads (Thermo Fisher, Waltham, MA, USA) at a speed of 4,000 RPM for 30 s using a Beadbug Microtube homogenizer (Benchmark Scientific Inc., Sayreville, NJ, USA). Cells were beaten a total of three times with incubation on ice for at least 30 s between pulses. Total protein concentration of the cell lysates was determined with Bradford reagent and spectrophotometry, and was used to normalize the LacZ activity. This was measured by incubation of cell lysates (at least three replicates per strain) at 37 °C with o-nitrophenol and was presented as Miller units ([OD_420_/mg protein * time in minutes] * 1,000). Statistical significance was examined using *t*-tests, two-tailed distributions, assuming equal variance, with a *P* < 0.05 considered significant.

To evaluate the *nmpR*-dependent regulation of *mxan_4236* during hypoxic conditions, strains were first grown overnight in aerated cultures to log phase growth. The next day, cells were pelleted and then suspended in fresh CYE to an OD_600_ equivalent of 2.2. Then, 1.5 mL of the concentrated cells was placed in a 2.0 mL tube with a gasket-sealed cap, similar to experiments that have been used elsewhere to evaluate hypoxic conditions (46). The sealed tubes were rocked at room temperature to prevent the *M. xanthus* strains from clumping and settling to the bottom of the tube. In this condition, the tubes became hypoxic in ~5 h as as evident in the clearing of methylene blue that was added to the control tubes as an indicator. After 20 h, individual tubes (*n* = 3, per strain) were processed identically as described above to determine LacZ production.

### Characterization of NmpU variants

NmpU and the NmpU^H96A^ proteins were purified as described above, with the addition of 300 µM hemin in dimethyl sulfoxide (DMSO) during cell lysis to maximize heme-containing NmpU molecules (35). The absorbance spectrum of the NmpR variants as purified was measured from wavelengths of 250–700 nm (NanoDrop 2000, Thermo Scientific). Absorbance over the entire spectrum was normalized for each protein to the absorbance at 280 nm and was plotted together as relative absorbance.

Autophosphorylation was carried out as previously described (30, 45). Resulting proteins were diluted to 50 µM in dialysis buffer. A 5 µL aliquot of the stock SK was added to 5 µL 10× kinase solution (250 mM Tris, pH 7.6, 10 mM MgCl_2_, 10 mM MnCl_2_, 10 mM CaCl_2_, 500 mM KCl, and 10 mM β-mercaptoethanol) and 35 µL of water. After 5 min of the protein equilibrating in the reaction buffer, an ATP mix (3 µM ATP-γ-^32^P [PerkinElmer, Waltham, MA, USA], 250 µM ATP) was added to the protein to initiate autophosphorylation. During incubation at room temperature, aliquots were removed at the indicated time points over 2 h at which point the reaction was stopped by adding SDS loading buffer (final concentrations: 50 mM Tris-HCL, pH 6.8, 2% SDS, 10% glycerol, 1% β-ME, 12.5 mM EDTA, and 0.02% bromophenol blue). Proteins were resolved on a 12% SDS-PAGE. The resulting gels were exposed to the same phosphor screen overnight and then visualized on a Molecular Dynamics Storm 860 imaging system. Resulting images were analyzed using ImageQuant (Molecular Dynamics) image quantification software. To determine the pixel density of the phosphorylated proteins, a box of consistent size was applied to the local pixel background and to each protein band. A representative image is shown. Quantification of the autophosphorylation is an average of three independent assays. The data are presented as the average signal intensity of each protein at each time point compared to wild-type NmpU at 120 min, which was set to 100% activity.

### Development and motility assays

*M*. *xanthus* strains were grown overnight to log phase. Cells were then washed with MMC buffer (20 mM MOPS, pH 7.6, 4.0 mM MgSO_4_, 2 mM CaCl_2_), suspended in MMC buffer to an OD_600_ equivalent of 2.2, and then replicates were plated as 10 µL spots on standard nomenclature (CF) starvation agar to induce fruiting body development (47). For the social motility assay, which was performed in conjunction with the development assay, 10 µL replicates of each strain were spotted in the center of 0.5% CYE agar plates (48). At least three biological replicates per strain were quantified. For both assays, all spots were allowed to dry, then plates were incubated at 32°C. Representative images of each strain were taken every day for 5 d with a Nikon SMZ1500 microscope, model C-DSD115, with a Lumenera Infinity 1 camera. Social, T4P-dependent motility was quantified by daily measurements of colony diameter over 5 d. The gross rate of motility was determined by plotting each replicate’s diameter over time, determining the millimeter-per-day colony expansion, and taking the average of the rates with Excel. Images shown are representative of at least three independent assays.

## RESULTS

### NmpR binds to a consensus sequence upstream of *pilR* (*mxan_5784*) and *mxan_4236*

We have previously demonstrated that NmpR binds with high specificity to promotor regions upstream of *pilR* (*mxan_5784*) and *mxan_4236*, the first gene in a putative operon that contains *nmpR* (30). Using these known DNA regions, we took a systematic approach to predict and define the consensus NmpR-binding sequence, and then used that sequence to identify the NmpR regulon.

NmpR is an NtrC-like RR. Members of this family of RRs typically bind an enhancer region containing two half sites bound by a dimer. However, unique to this family of RRs is oligomerization into functional hexamer or heptamer conformations that bind multiple enhancer sites (typically two to three sites per promoter) (49). Another important defining feature of NtrC-like RRs is their interaction with RNA polymerase holoenzymes containing the alternative σ^54^ sigma factor. Unlike the σ^70^-containing RNA polymerases, the σ^54^-containing RNA polymerase cannot initiate transcription intrinsically, and so it relies on interactions with NtrC (or an NtrC-like RR) (50). Therefore, an NtrC-like regulated promoter (i.e., a promoter that NmpR binds) would be predicted to have at least two enhancer sites and a σ^54^-binding site. To find sequences that would match these parameters, we used known NmpR-binding DNA fragments as inputs in the search FAIR: Finding All Identical Repeats (42). We focused on repeats that were five to seven nucleotides in length separated by approximately five nucleotides, consistent with the spacing of two half sites bound by NtrC-like RRs (51–54), and were found multiple times in both the *pilR* and *mxan_4236* promoter regions. The top hit that met these criteria was a repeat sequence of GCGCA that when inverted at one half site results in a sequence of GCGCA-N_5_-TGCGC (Fig. 2B and 3A, respectively).

To establish that this sequence was the binding site of NmpR *in vitro*, we used an EMSA using truncated DNA probes that either contained one, both, or neither putative site. NmpR bound to fragments of the *pilR* promoter when both binding sites were present (Fig. 2, Probe B, C, and E). This binding resulted in a step-wise shift, consistent with previous studies elsewhere that is indicative of different oligomerization states, with dimers shifting less relative to higher-order oligomers when there is a greater concentration of the protein (55). However, NmpR failed to bind to probes that were shortened to lack one or more of the sites (Fig. 2, Probes A and D). This observation is further supported by the lack of NmpR binding when the GCGC repeats of S2 were mutated to adenines to alter the sequence but preserve nucleotide spacing (Probe ES2*; for all promoters from here forward, the NmpR-binding site distal to the σ^54^ site and start codon is referred to as S1 and the proximal site is S2). Mutation of the putative σ^54^ site to adenines did not abolish NmpR binding, as predicted (Probe Eσ^54^*).

The results of NmpR binding to the *pilR* upstream region were reproducible with the *mxan_4236* promoter (Fig. 3). First, NmpR bound and shifted the WT *mxan_4236* probe and a σ^54^ mutated probe with the characteristic step-wise shift. Then, to better define the contribution of S1 and S2 in NmpR binding, we mutated the GCGC repeats in S1 or S2, or in combination, to adenines. When S1 was mutated (S1*), NmpR retained the ability to bind the probe, but a reproducible “smeared” shift was observed, which was suspected to be due to unstable oligomers forming when only S2 is accessible for NmpR binding. In contrast, when S2 was mutated (S2*), or when both sites were mutated in combination, no shift was observed, indicating that S2 is necessary for NmpR binding. The *pilA* promoter was included as a negative control, and NmpR did not bind this promoter, as previously established (30).

### The “ON” variant NmpR^V87E^ displays enhanced promoter binding

The Nmp pathway was discovered by identification of suppressor mutations that led to a constitutively active form of NmpR. One of the identified *nmpR* mutations leads to an amino acid substitution of valine to glutamic acid at position 87 in the protein sequence (V87E), which is referred to, going forward, as NmpR^ON^. Because NmpR^ON^ was predicted to be constitutively active, we purified this protein variant to assay its DNA-binding activity relative to the wild-type NmpR protein (Fig. 3B). The NmpR^ON^ variant shifted the WT *mxan_4236* promoter at the same concentrations as WT NmpR, suggesting that baseline DNA-binding affinity is not appreciably different between these two variants. However, NmpR^ON^ shifted the *mxan_4236* probe higher in the gel, consistent with a higher-order oligomerization state. This oligomerization difference of the NmpR^ON^ variant was even more pronounced when observing probes in which S1 and/or S2 were mutated. While the wild-type NmpR shifted S1* with a smeared pattern, the NmpR^ON^ variant maintained the step-wise pattern. Furthermore, NmpR^ON^ maintained the ability to shift the S2* probe, again in a step-wise manner.

**Fig 4:**
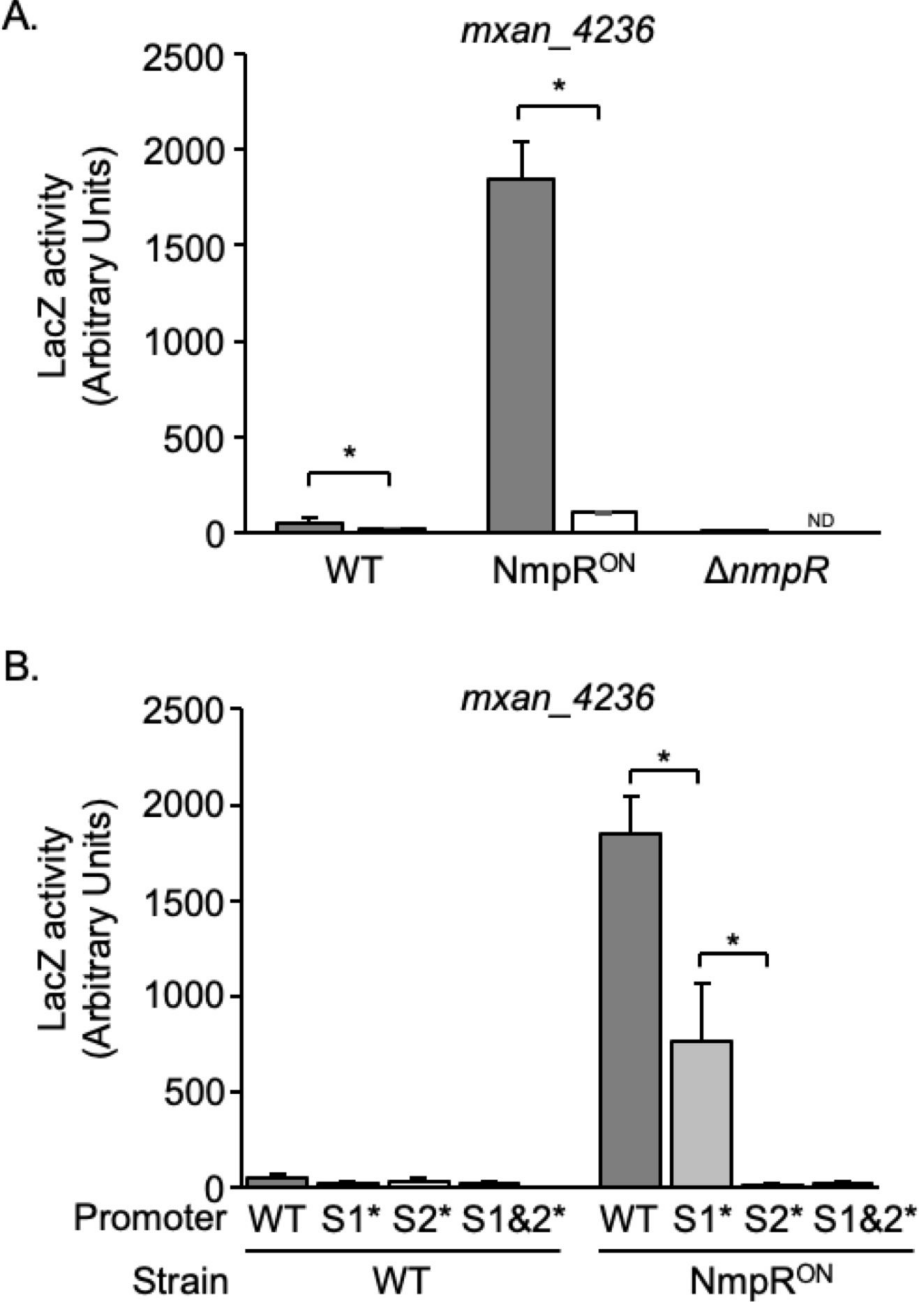
Expression from the *mxan_4236* promoter is NmpR dependent. (**A**) The *mxan_4236* promoter region (Fig. 3) was cloned upstream of a promoterless *lacZ* and integrated as a single copy into the indicated *M. xanthus* strains. LacZ activity was quantified from whole cell lysates following growth to log phase in broth culture with vigorous shaking to maintain aeration. Expression of *mxan_4236* was significantly upregulated in the NmpR^ON^ strain, and the σ^54^ site was necessary for expression (white bar). (**B**) When the S1 was mutated, expression of *mxan_4236* was significantly reduced in the NmpR^ON^ strain, and expression was absent when S2, or S1 and S2 in combination, was mutated. *P < 0.5; ND, not determined.

After observing this apparent increased oligomerization of the NmpR^ON^ variant, we repeated the assay with independent EMSAs using the wild-type *mxan_4236* promoter and a variant of NmpR that cannot be phosphorylated (D54A) or the phosphomimetic variant (D54E) (Fig. 3C). The wild-type NmpR and the NmpR^D54A^ behaved similarly in this assay, recapitulating again the step-wise shift. This demonstrated that the wild-type NmpR in this assay is likely unphosphorylated, and the shift is representative of the baseline NmpR-binding ability in this inactive state. On the other hand, the phosphomimetic NmpR^D54E^ has a higher-order shift at nearly all of the concentrations, and the NmpR^ON^ (V87E) shifts similarly. Collectively, this suggests that NmpR^ON^ has an increased propensity for oligomerization relative to the wild-type NmpR. The enhanced NmpR^ON^ oligomerization may make the protein sticky in this *in vitro* assay, a possible explanation for the present but reduced binding affinity and shift observed with the S1&S2* probe and the *pilA* negative control probe (Fig. 3B). While the exact oligomerization state of NmpR^ON^ in this assay cannot be directly ascertained, the observed difference in the pattern of the shift correlates with *in vivo* expression from the *mxan_4236* promoter (Fig. 4), demonstrating this variant is functionally active in the cells.

### NmpR^ON^ increases *mxan_4236* transcription and is dependent of the NmpR-consensus sequence and σ^54^-binding site

Having established that NmpR binds with specificity to a consensus sequence of GCGCA-N_5_-TGCGC in the *pilR* and *mxan_4236* promoters, we sought to correlate DNA binding *in vitro* with transcriptional regulation *in vivo* via the *mxan_4236* promoter. To demonstrate transcriptional regulation, the upstream region of *mxan_4236* was cloned in front of a promoterless *lacZ* on a single-copy plasmid integrated into the *M. xanthus* genome in either wild-type *M. xanthus*, an NmpR^ON^ strain, or a Δ*nmpR* strain. To assay the expression of the *lacZ* reporter, these strains were grown to log phase growth in nutrient-rich CYE broth at 32°C with shaking to aerate the cultures, lysed by bead beating, and assayed for β-galactosidase activity (Fig. 4). Basal expression from the *mxan_4236* promoter was very low in the wild-type strain (70 Units). However, when the σ^54^-consensus sequence was mutated, the expression level decreased to background, indicating the σ^54^ site is necessary for expression. In the NmpR^ON^ strain, the expression from this promoter was significantly increased 24-fold (1684 Units) compared to the wild-type strain. Even with this robust increase in *lacZ* expression in the NmpR^ON^ strain, the σ^54^-binding site was still necessary, indicated by the significant reduction in expression when this site was mutated (Fig. 4A). Finally, in the Δ*nmpR* strain, *lacZ* expression from the *mxan_4236* promoter was completely abolished. Overall, this data demonstrated several important observations that to date had only been inferred: *mxan_4236* is expressed at low amounts under standard aerated conditions, the NmpR^ON^ strain has high NmpR-dependent activity as indicated by the significant expression of *mxan_4236* in this strain, NmpR positively regulates its own promoter, and this transcriptional regulation requires the σ^54^ site for expression from this promoter.

To further investigate the regulation of NmpR at this promoter, the same S1 and S2 mutations introduced in the EMSA experiments were recapitulated in the *lacZ* reporter construct and integrated into the wild-type *M. xanthus* and the NmpR^ON^ strain. Regardless of the promoter construct, transcription from the *mxan_4236* promoter was near background levels in wild-type *M. xanthus*, further confirming that under the conditions in which we tested, the expression of *mxan_4236* is very low (Fig. 4B). By contrast, the expression from the *mxan_4236* promoter is substantially higher in the NmpR^ON^ strain compared to the wild-type strain. This expression was reduced significantly approximately twofold when S1 was mutated, and was abolished when S2, or S1 and S2 in combination, was mutated. These data demonstrate that the positioning of NmpR binding is critical. Even though NmpR^ON^ can bind when only S2 is available (Fig. 3B), interaction with the more distal S1 is important for optimal expression *in vivo* (Fig. 4B). On the other hand, when S2 is mutated, NmpR is still capable of binding to the promoter at S1 (Fig. 3B), but this binding is not sufficient for transcription initiation. Additionally, the residual binding of NmpR^ON^ to the promoter when both sites were mutated (Fig. 3B) is not sufficient for transcription (Fig. 4B).

Overall, the transcriptional analysis demonstrates that NmpR binding to the *mxan_4236* promoter is necessary for initiating transcription *in vivo*. The data show that while S1 is important for overall stabilization of NmpR at the promoter, it is S2 that is more proximal to the σ^54^ site, which is necessary for transcription.

### NmpR regulates several operons related to oxygen utilization

Having identified a necessary NmpR-binding'consensus sequence (GCGCA-N_5_-TGCGC) upstream of *pilR* and *mxan_4236*, we next searched the *M. xanthus* genome for sequences that matched the consensus using Pattern Locator (43). To have increased confidence in only true NmpR-binding sites in gene promoters, we used the following strict rules: (i) we allowed only two deviations from the consensus sequence per binding site, (ii) the distance between identified NmpR-binding sites had to be 5–50 bp from the next nearest binding site; the established sites upstream of *mxan_4236* are 14 bp apart and several NtrC-like RRs are 10–15 bp apart (49, 51–54), (iii) the distance between the NmpR-binding site and the nearest σ^54^-binding site had to be less than 500 bp; most NtrC-like RRs act within 100 bp of the σ^54^ (49), (iv) both the putative NmpR-binding sites and the σ^54^-binding site had to be in the same orientation of transcription with the next nearest gene, and (v) the σ^54^ site had to be less than 400 bp from the start codon of that gene.

**Fig 5:**
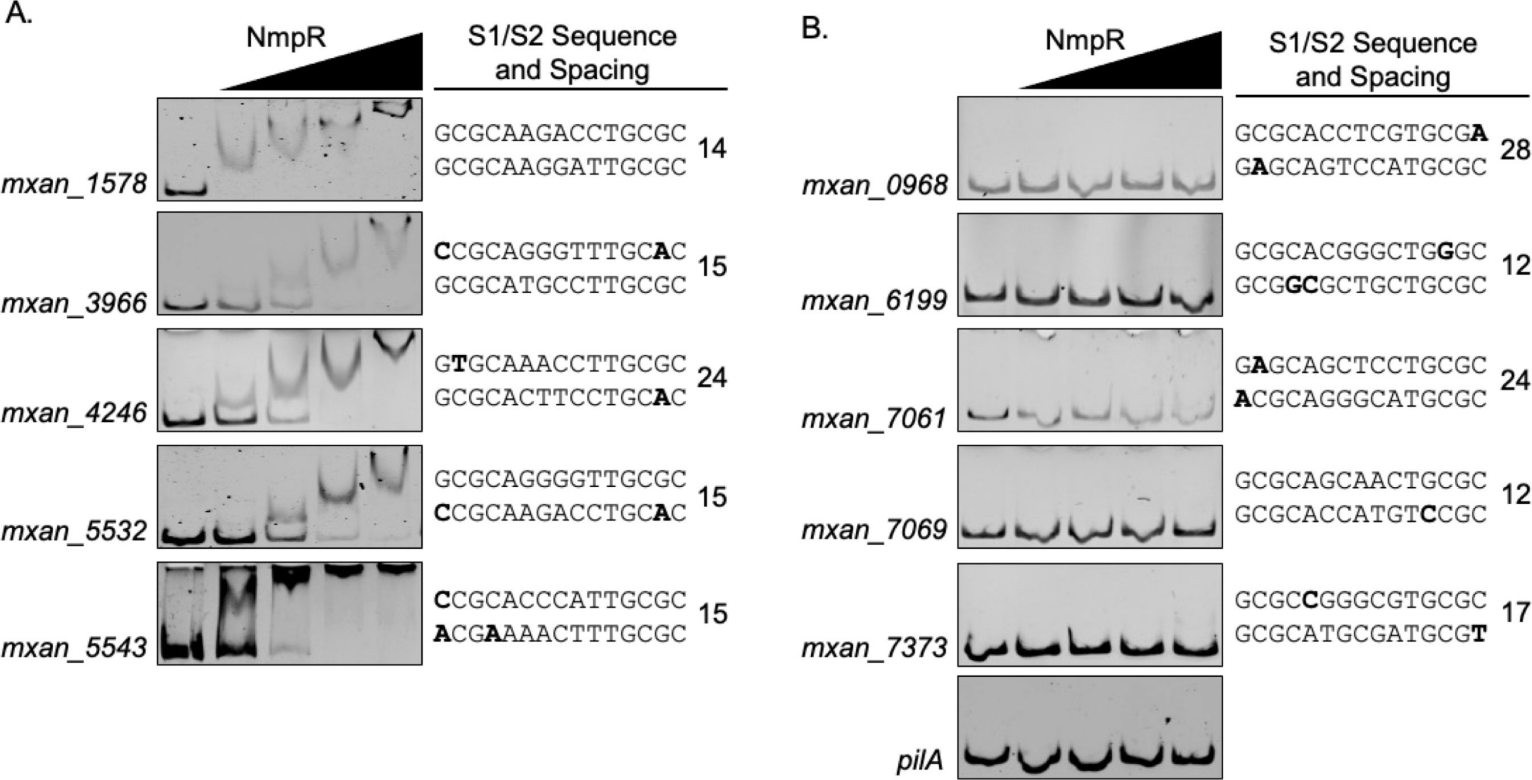
NmpR binds to the GCGCA-N_5_-TGCGC consensus sequence with high specificity to newly identified promoters. Searching the *M. xanthus* genome for the NmpR-consensus sequence predicted 10 additional promoters. NmpR bound to five of these promoters (**A**) but did not bind to others (**B**), indicating that the NmpR binding is specific. Locations in each of the S1 or S2 that diverged from the consensus appear in bold, and the spacing between each site is indicated next to those sequences for a given gene promoter. All the bound promoters had at least one site that was a perfect match for the consensus or diverged by only 1 bp. In contrast, unbound promoters all had at least one mismatch in each site. Additionally, those sequences bound by NmpR have a preferred spacing of 14–15 bp, while those that remained unbound had a sporadic spacing.

Based on the above criteria, 10 additional promoters were identified. Importantly, but as expected, the *mxan_4236* and *pilR* promoters were included in this data set. Of note, the sequence upstream of *nmpU* (*mxan_4246*) also met the criteria. The *nmpU* gene is the first gene in the likely operon that includes *nmpS* and *nmpT*. To establish if NmpR regulates these genes/operons, we used our *in vitro* and *in vivo* tools of EMSAs and *lacZ* reporters, respectively. DNA probes corresponding to each predicted region were generated and used as probes in the EMSA. Somewhat to our surprise, of the 10 newly identified regions, only five demonstrated binding by NmpR (Fig. 5). Consistent with the binding and oligomerization of NmpR, all of these probes displayed a similar step-wise shift pattern. For those probes that NmpR did not bind, we considered two parameters that may explain the lack of binding: the spacing between S1 and S2, and the divergence from consensus. Of the seven total promoters that NmpR bound in the EMSAs (*pilR*, *mxan_4236,* and the five newly identified promoters), five had a spacing between S1 and S2 of 14–15 nucleotides. The exceptions were *pilR* that has a spacing of five nucleotides, and *mxan_4246* that has a spacing of 24 nucleotides. In contrast, for those promoters that NmpR did not bind, the spacing was inconsistent (12, 12, 17, 24, and 28 bp), suggesting the spacing between the sites is important. Additionally, the total divergence from the consensus and the location of that divergence mattered. Of the NmpR-bound sequences, 6/7 had at least one site that contained a perfect match to the consensus at the 5′ end of that sequence. Additionally, 4/7 had at least one sequence that was a perfect GCGCA-N_5_-TGCGC match, and all the binding sites were a perfect match at the GCA-N_5_-TGC portion. In contrast, nearly all the sequences that NmpR did not bind had mismatches in at least one half site per repeat and/or were imperfect matches with the GCA-N_5_-TGC portions. Finally, looking again at the predicted sequences, we observed that of those that NmpR did not bind, 4/5 occurred in intragenic regions (i.e., the sequence was located in the nearest upstream gene), whereas 5/7 of the NmpR-bound sequences occurred in intergenic regions between genes. Collectively, this analysis emphasizes that NmpR binds with high specificity to its consensus sequence and has a preference for binding promoters in intergenic regions under the conditions of our assay.

**Fig 6:**
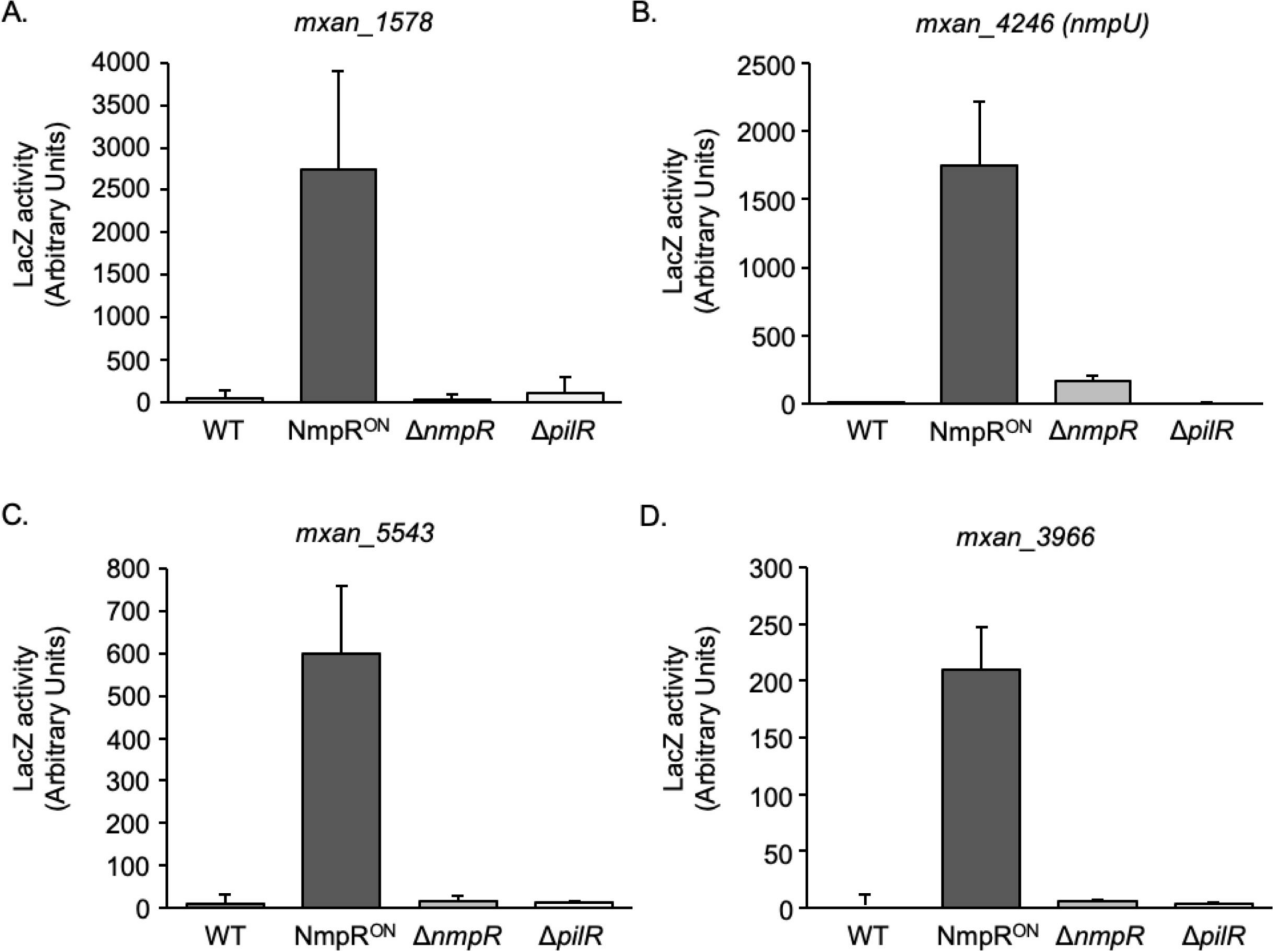
Expression of genes from the identified NmpR regulon is dependent on NmpR activity *in vivo*. LacZ reporter fusions were constructed for four of the genes identified by NmpR binding. All genes tested (**A–D**) were upregulated in an NmpR-dependent fashion. LacZ activity was normalized in the same manner, so the levels of expression can be compared between each in this figure, as well as the expression of *mxan_4236* in Figure 4. Note that the scale of the y-axis is different for each graph to emphasize the pattern of expression, although the amplitude of expression is different.

**Fig 7:**
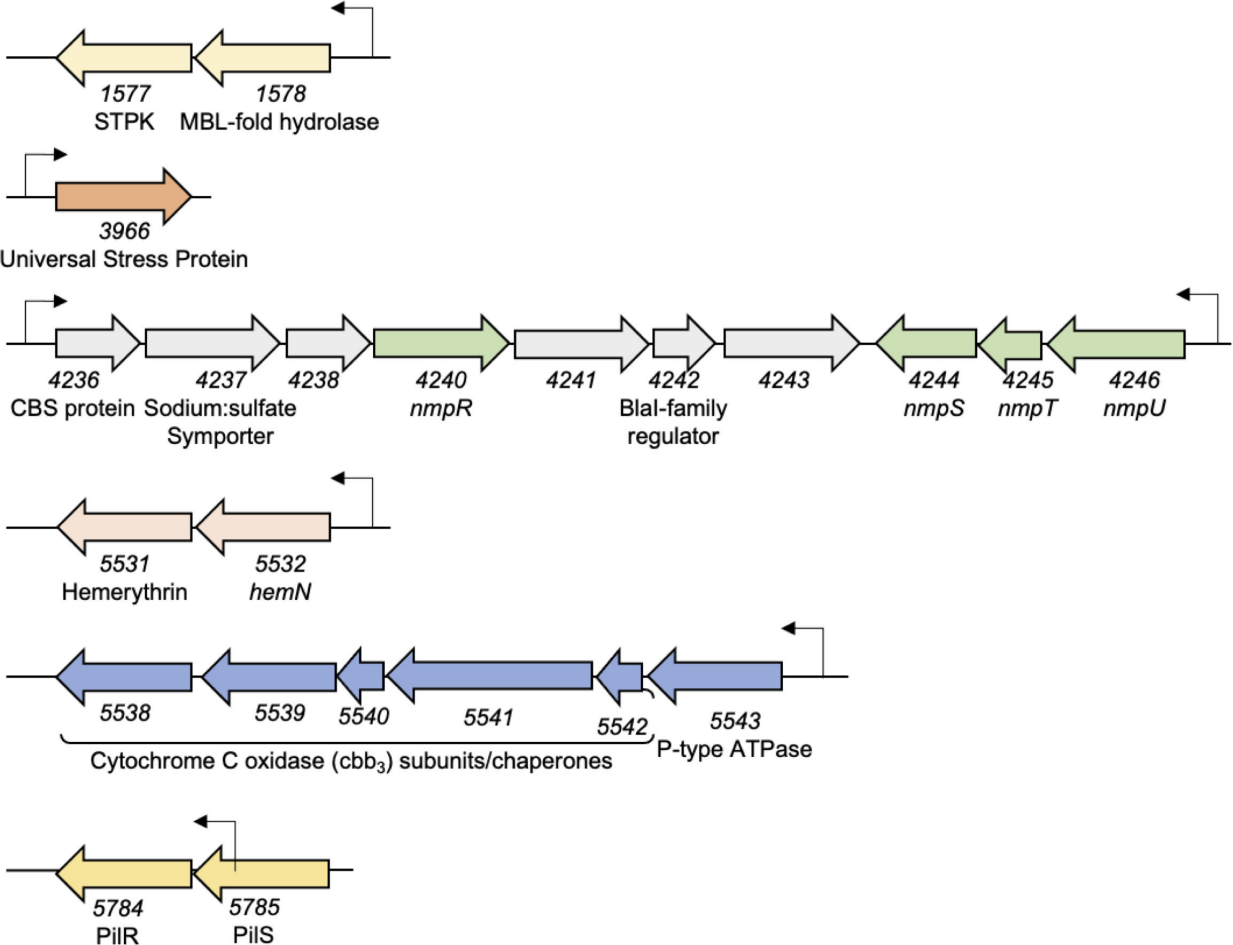
The identified NmpR-dependent regulon. All the genes in each putative operon regulated by NmpR are shown. Gene numbering is based on the *M. xanthus* DK1622 annotation (11). Promoters are indicated with an arrow extending above each operon. For the operons themselves, the genomic orientation and size of each gene are drawn to scale.

Finally, promoters that displayed NmpR binding correlated with the upregulation of those genes in the NmpR^ON^ strain (Fig. 6). Although the magnitude of expression from these promoters was varied, each displayed the same pattern of expression seen for *mxan_4236* (Fig. 4), with a clear upregulation of each gene in the NmpR^ON^ strain. In contrast, the expression of the genes tested was at background levels in the wild-type *M. xanthus* and Δ*nmpR* strains, indicating the expression is NmpR dependent under the conditions we tested. As an added control for this experiment, we also looked for expression of these genes in a Δ*pilR* strain, because the background genotype of the NmpR^ON^ strain is Δ*pilR* (30). There was no expression of any of the genes in the Δ*pilR* strain, indicating the increased expression is solely dependent on the activity of NmpR and not influenced by the lack of PilR in those strains. Overall, our *in silico* prediction coupled with EMSAs and expression analysis *in vivo* has collectively identified an NmpR-dependent regulon consisting of seven promoters and a total of 23 total genes based on genomic organization and predicted operon structure (Fig. 7).

### The activity of NmpU, the initial SK in the NmpRSTU pathway, requires heme binding

**Fig 8:**
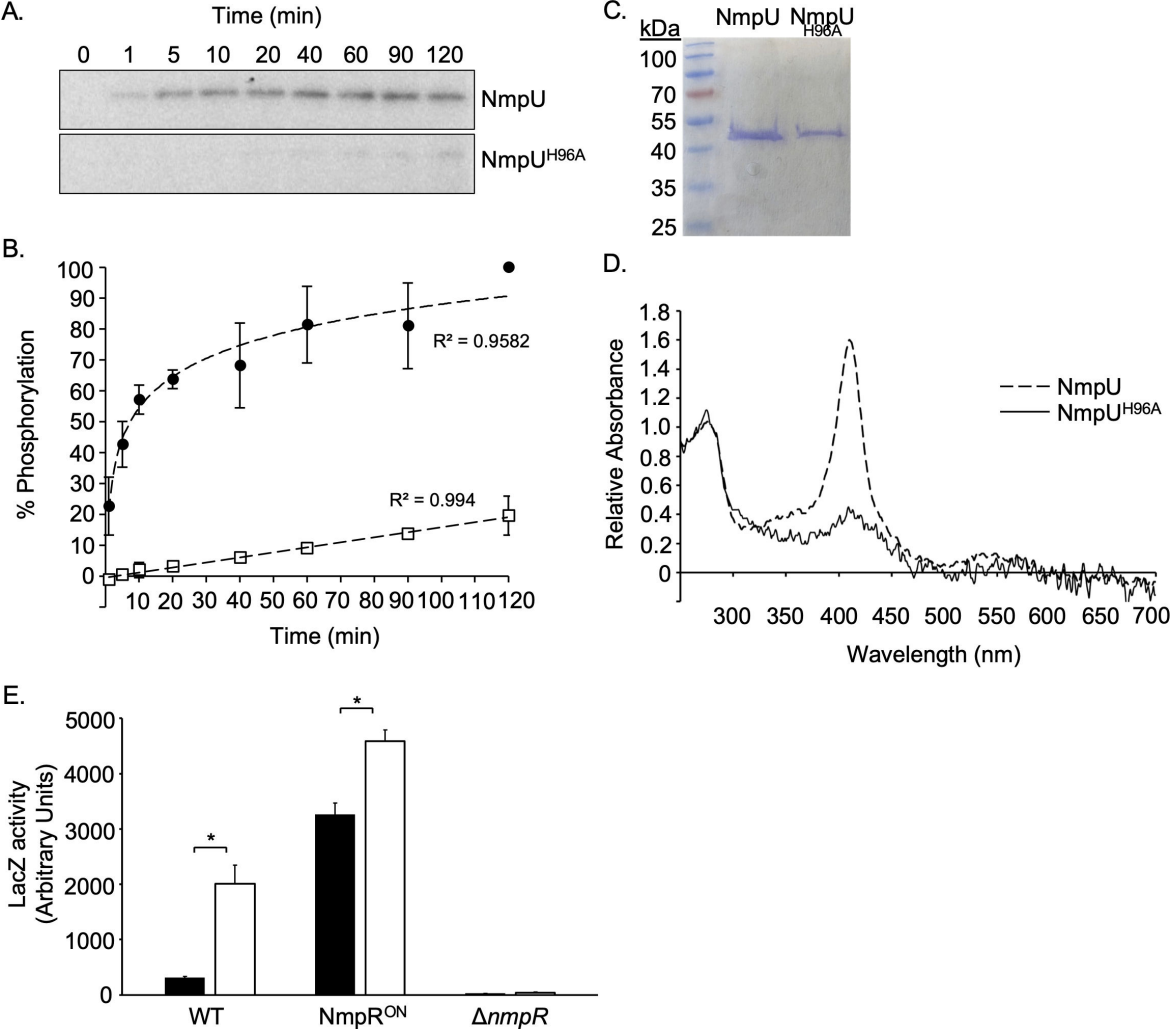
Heme binding is necessary for NmpU activity, and NmpR upregulates *mxan_4236* during hypoxia. (**A**) NmpU and a variant unable to bind heme, NmpU^H96A^, were both purified and used in an autophosphorylation assay. A representative time-course of the assay is shown. (**B**) The accumulation of phosphorylation by each protein was determined (*n* = 3) by measuring the pixel density at each time point, with the NmpU signal at 120 min set as 100% activity. (**C**) Both protein variants used in this assay were qualitatively assessed for purity by Coomassie staining of each protein as purified. (**D**) The absorbance spectrum of NmpU and NmpU^H96A^ was normalized to the absorbance at 280 nm of each protein individually and was plotted together. The absence of a peak at ~410 nm indicates the NmpU^H96A^ variant does not bind heme. (**E**) The LacZ reporter fusion strains used in Figure 4 were grown first in aerated cultures overnight, then concentrated and placed in individual gasket-sealed tubes to induce a hypoxic state. After 20 h, individual tubes were opened and processed as previously stated for LacZ activity (*n* = 3). Asterisk indicates a significant difference (unpaired *t*-test, *P* < 0.05).

*Anaeromyxobacter* sp. Fw109-5, a closely related species of *M. xanthus*, contains all the homologs of the NmpRSTU system, and the *Af*GcHk protein, specifically, is homologous to NmpU. *Af*GcHk utilizes its protoglobin domain to sense oxygen, and a loss of heme binding significantly reduces its autophosphorylation rate (32, 33, 35). To establish that *M. xanthus* NmpU activity is consistent with that of *Af*GcHk, a variant of *M. xanthus* NmpU was constructed that changed a histidine to an alanine at amino acid position 96 (H96A). This variant was predicted to cause NmpU to lose its ability to bind heme, as this histidine is part of the heme ligand-binding site (32, 33, 35). Wild-type NmpU and NmpU^H96A^ purify identically (Fig. 8C) and, importantly, NmpU^H96A^ was unable to bind heme, as evident in the loss of a prominent absorbance peak at ~410 nm (Fig. 8D) (35). These purified proteins were then subjected to an autokinase assay (Fig. 8). NmpU autophosphorylation accumulated rapidly, with an activity observed within 1 min. Saturating autophosphorylation was observed in this assay at ~1 h of incubation. In contrast, the NmpU^H96A^ autophosphorylation rate was dramatically reduced and never reached saturation over the 2 h of assay. This indicates that heme binding is necessary for NmpU autophosphorylation, consistent with its predicted role as an oxygen sensor.

### Expression of *mxan_4236* is upregulated under hypoxic conditions and is NmpR dependent

Given the heme-dependent activity of NmpU and the potential function of the NmpR-dependent regulon, we sought to evaluate the expression of *mxan_4236* under hypoxic conditions. To induce a hypoxic state, strains were initially grown overnight in aerated cultures to log phase growth. The next day, the cells were concentrated, and 1.5 mL of the concentrated cells was placed in 2.0 mL tubes with gasket-sealed caps. This assay was based on a well-described rapid dormancy model used in *Mycobacterium tuberculosis* research and relies on the fact that the cells in the tubes will rapidly consume the oxygen, inducing a hypoxic or anaerobic condition (46). The loss of oxygen is indicated by the clearing of methylene blue. Indeed, for each *M. xanthus* strain, indicator tubes with methylene blue added turned from a blue/green color to clear in ~5 h. To quantify the impact of this hypoxic state on *mxan_4236* expression, LacZ production was measured after 20 h in the sealed tubes (Fig. 8E). The expression of *mxan_4236* was significantly greater after incubation in this hypoxic state in the wild-type strain (6.5-fold) and, as seen before, the expression in the Δ*nmpR* strain was at background levels. Expression was also significantly greater in the NmpR^ON^ strain after 20 h, although the magnitude of the increase was less (1.4-fold). Collectively, this suggests that gene regulation of the NmpRSTU system is in response to a reduced oxygen concentration and is consistent with the heme-dependent activity of NmpU.

### Activation of the NmpRSTU pathway restores motility to *M. xanthus* Δ*pilR* but does not restore fruiting body development

**Fig 9:**
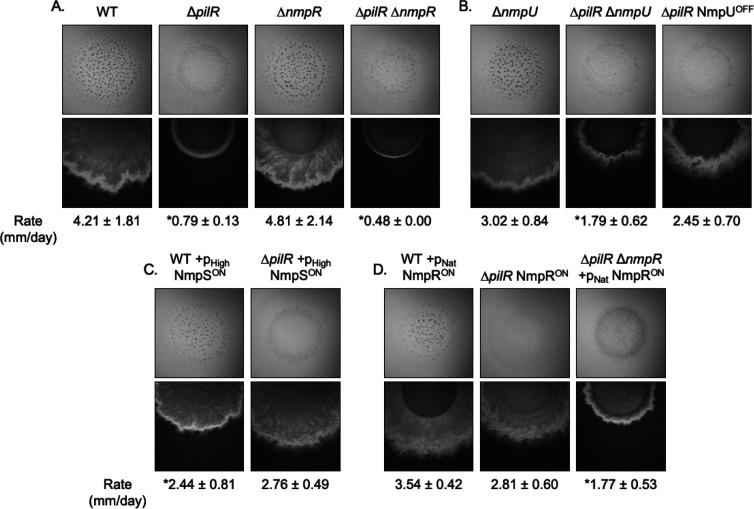
NmpR^ON^ mutations restore T4P-dependent motility in a ∆*pilR* background but not fruiting body development. (**A**) The wild type develops numerous mature fruiting bodies and displays robust T4P-dependent motility on soft agar, as evident in the flared colony edges. The ∆*pilR* strain fails to develop and does not display T4P-dependent motility, which is as evident in the clean, delineated colony edge. The small measured rate of expansion in the nonmotile strains is due to the replication of the cells in the colony. The ∆*nmpR* strain has the same phenotype as the wild type, which is expected because NmpR is “OFF” in ambient laboratory conditions. (**B–D**) Expression of the indicated *nmp* genes was driven by either the promoter pHigh (623 bp upstream of *mxan_4894*, *groES*) or pNat (585 bp upstream of *mxan_4236*; the same promoter used throughout this paper), both of which have been used in previous studies for complementation (30). Strains with NmpR “ON” through mutation in *nmpU* (**B**), *nmpS* (**C**), or *nmpR* (**D**) in a ∆*pilR* background do not develop despite the restoration in T4P-dependent motility. In contrast, all NmpR^ON^ strains with a wild-type background displayed intact T4P-dependent motility and some fruiting body formation. All pictures depicted in this figure were taken after 4 d at 32°C and are representative of at least three biological replicates per strain. *Significantly different from wild type (unpaired *t*-test, *P* < 0.05).

The NmpRSTU pathway was discovered through suppressor mutations that restored T4P-dependent social motility (30). However, the role of NmpRSTU in other cooperative *M. xanthus* behaviors is unknown. We investigated whether the ability of an active NmpR to restore motility to the *M. xanthus* Δ*pilR* strain would also restore fruiting body development (Fig. 9). To attempt to limit variation between culture preparations, motility assays and development assays were carried out simultaneously, and a broad panel of strains was tested, representing multiple branch points that would culminate in an NmpR^ON^ state (i.e., by manipulation of *nmpU*, *nmpS*, or *nmpR*). Relevant controls for the assay were included to establish the background motility, or lack thereof, of each parental strain (Fig. 9A). The wild-type *M. xanthus* strain had an average colony expansion of 4.21 mm/day and development of numerous mature fruiting bodies over the course of 5 d, with the first fruiting bodies typically visible by the third day. As expected, the ∆*pilR* strain lacked T4P-dependent motility and did not develop fruiting bodies. The ∆*nmpR* strain displayed T4P-dependent motility and fruiting body development phenotypes similar to that of the wild type, consistent with NmpR being “OFF” in the presence of oxygen. Finally, the ∆*pilR*∆*nmpR* strain did not display T4P-dependent motility or develop fruiting bodies, identical to the ∆*pilR* phenotype.

To determine the impact of constitutive activity of NmpR in a ∆*pilR* background, each branch point of the Nmp pathway was manipulated to activate NmpR. Regardless of which gene in the Nmp pathway was mutated, social motility was restored, but fruiting body development was not (Fig. 9B through D). The gross rate of colony expansion, indicating rescued motility, varied between these strains, with some having rates comparable to wild type, while others demonstrated reduced motility rates. However, regardless of the motility rate, development was not restored to the Δ*pilR* strain. We also considered the impact of the Nmp pathway manipulations in the wild-type background strain that contained an intact and functional *pilR* (Fig. S1). In these strains, T4P-dependent motility was comparable to wild-type motility (Fig. 9). However, fruiting body development was delayed and incomplete after 5 d of observation (Fig. S1). Because of some variability in the phenotype, in some assays, fruiting body development in these otherwise wild-type strains was not observed at all (data not shown). Therefore, NmpR activity rescues the T4P-dependent motility of a ∆*pilR* strain, but that same NmpR activity is not sufficient to restore fruiting body development. Additionally, NmpR activity under oxygen-replete conditions in which NmpR is typically inactive, delays or inhibits development, suggesting a role for oxygen sensing in the timing of fruiting body formation.

## DISCUSSION

### NmpR binds to a defined consensus sequence, acting as a typical NtrC-like RR

We have demonstrated that NmpR is a typical NtrC-like RR and further provide evidence that the NmpRSTU signaling system is an oxygen-responsive system that influences *M. xanthus* development. NmpR specifically binds to enhancer sites composed of a consensus sequence of GCGCA-N_5_-TGCGC. This dyad-symmetrical sequence is the typical pattern of NtrC-like RR-binding sites (51–53, 55), and our analysis continues to reaffirm this pattern of DNA binding and transcriptional regulation of this class of RR. NmpR binds to multiple enhancer sites in each promoter, and the binding appears cooperative and most efficient when NmpR is in an active state. For example, NmpR binds in a stepwise, concentration-dependent fashion to each promoter tested, indicative of higher-order oligomerization states. However, the mutation of S1 in the *mxan_4236* promoter (forcing binding to S2 exclusively) resulted in a smeared pattern of the DNA shift. This has been observed elsewhere and suggests unstable oligomers (55). However, the smear was not observed with the NmpR^ON^ variant, suggesting more stable oligomers. This is further supported by the observation that the NmpR^ON^ variant binds in a pattern consistent with the NmpR^D54E^ phosphomimetic variant. The importance of an active NtrC oligomer (via phosphorylation or by mutation) to the formation of a multimeric complex has also been confirmed by other labs (49, 55–58). The pattern of transcriptional regulation of *mxan_4236* and the newly identified NmpR-dependent genes (discussed below) verifies that NmpR upregulates these genes when NmpR is in an active conformation. Furthermore, in our previous study, only complementation with NmpR^V87E^ or NmpR^D54E^ was sufficient to restore motility to the Δ*pilR*Δ*nmpR* strain, while complementation with wild-type NmpR or NmpR^D54A^ was not (30).

### The NmpRSTU system likely responds to oxygen depletion and regulates an oxygen utilization operon

We have previously modeled that NmpU serves as the master sensor of the NmpRSTU system by sensing oxygen via its protoglobin domain (Fig. 1). This model is further supported by the demonstration that NmpU requires heme binding for autophosphorylation (Fig. 8). While this does not directly demonstrate that the heme is sensing oxygen, it is strongly suggestive given that the NmpU homolog in *Anaeromyxobacter* sp. Fw109-5 requires heme and is active in the presence of oxygen (32–35). Interestingly, *Anaeromyxobacter* is capable of anaerobic growth, a condition in which the Nmp system would be typically in an ON state for gene regulation. Currently, it is unknown what genes the Nmp system regulates in *Anaeromyxobacter*, but we would predict that these two systems likely regulate shared genes but with some species-specific genes based on aerobic growth requirements of *M. xanthus*. Indeed, in *Anaeromyxobacter* sp. Fw109-5, the *M. xanthus* genomic organization of *nmpS*, *nmpT*, and *nmpU* is conserved, and the *nmpR* homolog is upstream of *nmpS* on the genome and is transcribed in the opposite orientation (Fig. 7). However, in *Anaeromyxobacter*, the remaining genes found in the *M. xanthus nmpR* operon (*mxan_4236–mxan_4243*) are absent, suggesting conservation of the Nmp pathway but some genomic rearrangements at this locus. Interestingly, it is suspected that the last common ancestor of *Anaeromyxobacter* and *M. xanthus* was an aerobic organism, which may explain the homology between the NmpRSTU system in these species (59). However, in contrast to *Anaeromyxobacter*, *M. xanthus* is widely regarded as an obligate aerobe so a failure to respond to changing oxygen concentration would likely result in cells being unable to produce factors needed for survival in reduced oxygen environments. Therefore, it is significant that the identified NmpR regulon contains several genes with clear connections to oxygen utilization.

The regulation of the *pilR* promoter by NmpR, and therefore the role in the regulation of T4P-dependent motility, remains unexplored. During this study, we attempted to use a *lacZ* reporter fusion to characterize transcription from this promoter. However, the NmpR-regulated promoter of *pilR* is atypical, especially compared to the clear intergenic promoters of the other NmpR-dependent genes. Instead, the promoter that NmpR would use to regulate *pilR* is contained within the upstream gene *pilS* and would lead to a transcript with a long 5′-untranslated leader sequence (Fig. 2 and [30]). Recently, and contrary to our observation of the NmpR-regulated genes, a description of the Nla28 operon in *M. xanthus* observed a majority of genes regulated by that NtrC-like RR to be in intragenic regions (60, 61). Those authors proposed that utilizing intragenic regions as promoters with long leader sequences likely provide opportunities for post-transcriptional regulation or for altering the relative concentrations of different genes within operons. This is consistent with our proposed model of NmpR regulating *pilR* transcripts to increase PilR concentration independently from its cognate SK, PilS, which is typically transcribed on a shared transcript (29). Future studies intend to investigate the regulation of *pilR* and the overall role of NmpRSTU on *M. xanthus* T4P-dependent motility, especially in the context of oxygen limitation.

Beyond *pilR*, the NmpR regulon has clear links to oxygen utilization. First, NmpR binds to and upregulates *mxan_4236*, the first gene in the operon that contains *nmpR* itself, and *mxan_4236* expression significantly increases under hypoxic conditions in an NmpR-dependent fashion (Fig. 8). Additionally, we identified an NmpR-dependent promoter upstream of *nmpU*, the first gene in a putative operon that contains the remaining components of the Nmp multi-component system (*nmpU*, *nmpT*, and *nmpS* in order of transcription). Therefore, all of the components of the Nmp system are regulated by NmpR. The first gene in the *nmpR* operon is *mxan_4236*, which encodes a CBS-domain protein. The CBS-domain protein family is abundant in all kingdoms of life, from bacteria and archaea to fungi, plants, and animals (62). The function of these proteins is varied, but a predominant functional theme of these proteins is to check the energy status of the cell by binding AMP, ADP, and/or ATP (62), a state that would certainly be altered in *M. xanthus* due to respiratory changes resulting from a decrease of oxygen availability. Interestingly, in *Anaeromyxobacter* sp. Fw109-5 immediately upstream of the *nmpU* homolog is a dual CBS-domain protein, suggesting that this type of protein may be important in the NmpR-dependent response across species.

Another gene regulated by NmpR, *mxan_3966* [MXAN_RS19280], encodes a single domain universal stress protein (USP). Like CBS-domain proteins, USPs such as MXAN_3966 are an ancient protein domain found across all domains of life and collectively have a wide range of functions (63). Additionally, like CBS-domain proteins, USPs have a well-conserved tertiary structure and based on solved structures often bind nucleotides such as ATP (63–69). MXAN_3966, regulated by NmpR, shares the consensus sequence for ATP binding (G-2X-G-9X-S/T), therefore MXAN_3966 may serve as another sensor of the energy state of the cell. This proposed function is consistent with the function of USPs from *Mycobacterium tuberculosis* that are upregulated by a dormancy regulon induced by hypoxia and play a role in the response to hypoxia stress (70–72), and is consistent with the role of USPs in *Pseudomonas aeruginosa* (73, 74), *Burkholderia* spp. (75, 76), and *Micrococcus luteus* (77).

An even clearer connection with oxygen utilization is demonstrated by the NmpR regulation of a P-type ATPase and the downstream genes encoding the high oxygen-affinity cytochrome c oxidase (*mxan_5543-mxan_5538* [MXAN_RS26835-MXAN_RS26860]). *M. xanthus* encodes six P-type ATPases, and three of them have been defined as having important roles in copper transporter by moving copper out of the cell to maintain copper homeostasis (78). Although MXAN_5543 was not characterized for copper transport in that study, P-type ATPases like MXAN_5543 are often associated with *cbb_3_* cytochrome c oxidase complexes like *mxan_5542-mxan_5538* and function to deliver Cu^+^ to the *cbb_3_* heme-copper oxidase complex (79, 80). The alternative electron transport of the Cbb_3_ oxidase family is distinct from the other respiratory pathways in that it utilizes heme-copper and functions under hypoxic conditions with a high affinity for oxygen. Bacteria utilize this oxidase to maintain respiration in reduced oxygen environments and typically regulate expression of this oxidase under those same hypoxic conditions, for example, via the FixLJ TCS or the transcriptional regulator Fnr (81–84). The regulation of the *cbb_3_* cytochrome c oxidase in *M. xanthus* by NmpR is consistent with this type of regulation.

Finally, NmpR binds the promoter of a putative two-gene operon with a link to oxygen utilization, *mxan_5532* [MXAN_RS26825] encoding HemN and *mxan_5531* [MXAN_RS26820] encoding a hemerythrin protein. HemN is an enzyme in the biosynthesis of heme, catalyzing the first of three terminal steps leading to the maturation of heme in the classical pathway. Notably, HemN enzymatic activity is oxygen independent (85, 86). Interestingly, *M. xanthus* has an alternative enzyme at this branch point in heme biosynthesis, the putative oxygen-dependent HemF (*mxan_6762*). In several species, HemF and HemN are differentially regulated in an oxygen-dependent fashion (87, 88). For example, *E. coli* has a single enzyme for each catalytic step of heme biosynthesis, with the notable exception of HemF and HemN. They are differentially regulated because HemN can function with or without oxygen, while HemF is increased to deal with oxidative stress (89). Similar HemN function and regulation have been observed in *Bacillus subtilis* (85, 90), *Pseudomonas aeruginosa* (91), and *Bradyrhizobium japonicum* (92). Downstream of *hemN* is *mxan_5531* encoding a single-domain hemerythrin protein. Hemerythrins are oxygen-binding proteins that have long been appreciated in multiple eukaryotes, as well as in many different species of bacteria (93–96). Hemerythrins bind oxygen reversibly via the interaction with two non-heme iron atoms coordinated by conserved histidine residues and aspartic acid (or glutamic acid) residues (93, 97). In bacteria, hemerythrins have been shown to bind oxygen for general oxygen sequestration or specific functions. For example, the methanotroph *Methylococcus capsulatus* uses single-domain hemerythrins to deliver oxygen to methane monooxygenases for the catabolism of methane (98). *M. xanthus* may utilize MXAN_5531 and other hemerythrins encoded in the genome to scavenge oxygen during conditions of limited oxygen concentration.

### NmpRSTU may play a role in *M. xanthus* fruiting body development

A significant remaining question regards the role of NmpR in regulating the cooperative behaviors of *M. xanthus* such as social motility, predation, and fruiting body development. Our results show that even the constitutive activity of NmpR cannot overcome the necessity for PilR in development, even though T4P-dependent motility is restored. Significantly, this is true across several strains that have been genetically modified to induce an NmpR^ON^ state. We have also shown that when the wild-type strain (with an otherwise functional PilR and NmpR) also has the Nmp system genetically turned ON, it is delayed for development. We speculate that this may be due to the functional consequence of continuous PilA production in the NmpR^ON^ state. Elsewhere it has been shown that *pilA* expression decreases during development (28), so when NmpR is constitutively active, it is perhaps that too much PilR and/or PilA accumulates and is detrimental to fruiting body formation and/or sporulation. Furthermore, transcriptomic analysis of developing *M. xanthus* populations demonstrated that several of the genes in the NmpR-dependent regulon are downregulated during different stages of development including *mxan_1578* [MXAN_RS07670] early in development, the *nmp* operons both early and late, and the *cbb_3_* P-type ATPase during sporulation (99). We also propose that limited oxygen may in fact be a signal to not form fruiting bodies, perhaps through energy production costs, as has been observed by reduced sporulation of *Bacillus* species during anaerobic conditions (100–103).

Overall, we propose that NmpRSTU is an oxygen-sensing system of *M. xanthus* and that it regulates genes that may be important for critical physiological responses to limited oxygen availability. We chose a strict approach to identify NmpR-regulated genes, and it is possible that transcriptomics may reveal further NmpR-regulated genes beyond this study. Future work will focus on defining oxygen-dependent phenotypes that *M. xanthus* is likely to require in the soil and the role of NmpRSTU and the genes of its regulon in regulating the multicellular behaviors of *M. xanthus* under those conditions.

## SUPPLEMENTAL MATERIAL

Figure S1 jb.00280-24-s0001.tiff Constitutive activity of NmpR delays development.

Tables S1 and S2 jb.00280-24-s0002.docx Table S1: *M. xanthus* strains and plasmids. Table S2: Primers used in this study.

ASM does not own the copyrights to Supplemental Material that may be linked to, or accessed through, an article. The authors have granted ASM a non-exclusive, world-wide license to publish the Supplemental Material files. Please contact the corresponding author directly for reuse.
