# Supplementary figures and images for: The NmpRSTU multi-component signaling system of Myxococcus xanthus regulates expression of an oxygen utilization regulon
Source: J Bacteriol. 2025 Jan 27;207(2):e00280-24. doi: 10.1128/jb.00280-24 (PMC11841059; doi:10.1128/jb.00280-24)

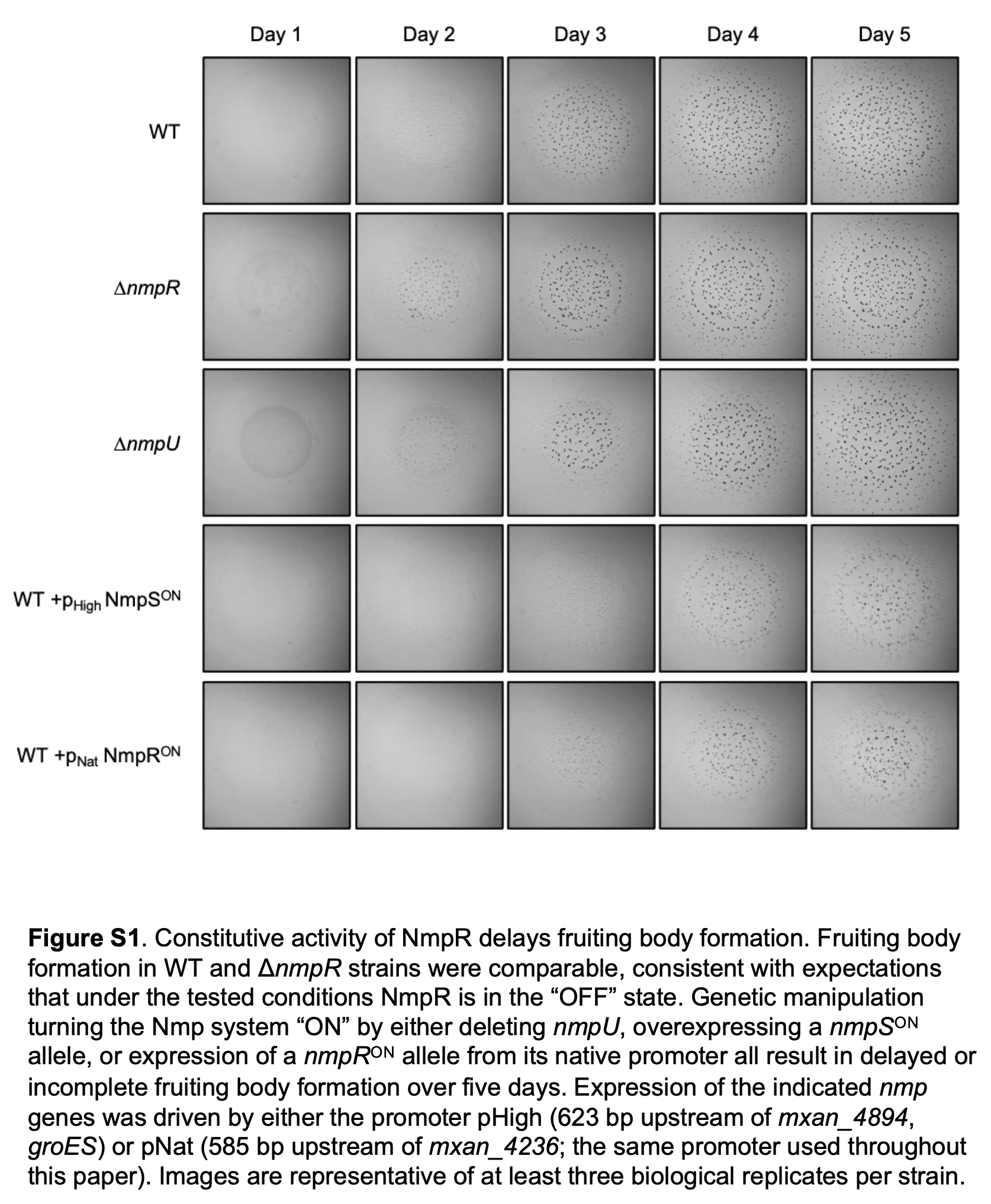

Supplement: Figure S1 — Constitutive activity of NmpR delays development. [file jb.00280-24-s0001.tiff]
