# Supplementary material for: The NmpRSTU multi-component signaling system of Myxococcus xanthus regulates expression of an oxygen utilization regulon
Source: J Bacteriol. 2025 Jan 27;207(2):e00280-24. doi: 10.1128/jb.00280-24 (PMC11841059; doi:10.1128/jb.00280-24)
Supplement: Tables S1 and S2 — Table S1: M. xanthus strains and plasmids. Table S2: Primers used in this study. [file jb.00280-24-s0002.docx]

Table S1. *M. xanthus* Strains and Plasmids^1^

| **Strain** | **Relevant genotype** | **Source** |
| --- | --- | --- |
| DZ2 | Wild-type | (1) |
| JK4281 | Δ*pilR* | (2) |
| JK4461 | Δ*pilR nmpRV87E*; NmpR^ON^ | (3) |
| JK4675 | Δ*nmpR* | (3) |
| JK4716 | Δ*pilR* Δ*nmpR* | (3) |
| JK4786 | Δ*pilR* Δ*nmpU* | (3) |
| JK4907 | Δ*nmpU* | (3) |
| JK4460 | Δ*pilR mxan_4246* C847T; NmpU^OFF^ | (3) |
| JK5082 | *M. xanthus* with pHigh-NmpS D59A; NmpS^ON^ | (3) |
| JK5086 | Δ*pilR* with pHigh-NmpS D59A; NmpS^ON^ | (3) |
| JK4846 | Δ*pilR* Δ*nmpR* with pNat-4240V87E | (3) |
| JK4882 | *M. xanthus* with pNat-4240V87E | This study |
| JK5228 | *M. xanthus* with pCD127-*mxan_4236pro* | This study |
| JK5255 | *M. xanthus* with pCD127-*mxan_4236pro-*S1* | This study |
| DB1067 | *M. xanthus* with *pCD127-mxan_4236pro-*S2* | This study |
| DB1061 | *M. xanthus* with *pCD127-mxan_4236pro-*S1S2* | This study |
| JK5269 | *M. xanthus* with *pCD127-mxan_4236pro-σ^54^** | This study |
| JK5277 | JK4461 with pCD127-*mxan_4236pro* | This study |
| JK5278 | JK4461 with pCD127-*mxan_4236pro-*S1* | This study |
| DB1070 | JK4461 with *pCD127-mxan_4236pro-*S2* | This study |
| DB1064 | JK4461 with *pCD127-mxan_4236pro-*S1S2* | This study |
| JK5280 | JK4461 with *pCD127-mxan_4236pro-σ^54^** | This study |
| JK2581 | JK4675 with pCD127-*mxan_4236pro* | This study |
| DB1125 | *M. xanthus* with pCD127-*mxan_1578pro* | This study |
| DB1127 | JK4461 with pCD127-*mxan_1578pro* | This study |
| DB1129 | JK4675 with pCD127-*mxan_1578pro* | This study |
| DB1131 | JK4281 with pCD127-*mxan_1578pro* | This study |
| DB1099 | *M. xanthus* with pCD127-*nmpUpro* | This study |
| DB1105 | JK4461 with pCD127-*nmpUpro* | This study |
| DB1111 | JK4675 with pCD127-*nmpUpro* | This study |
| DB1135 | JK4281 with pCD127-*nmpUpro* | This study |
| DB1104 | *M. xanthus* with pCD127-*mxan_5543pro* | This study |
| DB1109 | JK4461 with pCD127-*mxan_5543pro* | This study |
| DB1115 | JK4675 with pCD127-*mxan_5543pro* | This study |
| DB1117 | JK4281 with pCD127-*mxan_5543pro* | This study |
| DB1101 | *M. xanthus* with pCD127-*mxan_3966pro* | This study |
| DB1107 | JK4461 with pCD127-*mxan_3966pro* | This study |
| DB1113 | JK4675 with pCD127-*mxan_3966pro* | This study |
| DB1133 | JK4281 with pCD127-*mxan_3966pro* | This study |
|  |  |  |
| **Plasmid** | **Use** | **Source** |
| pET28a-NmpR | Full-length NmpR | (3) |
| pET28a-NmpRV87E | Full-length NmpR^V87E^ | This study |
| pET28a-NmpRD54A | Full-length NmpR^D54A^ | This study |
| pET28a-NmpRD54E | Full-length NmpR^D54E^ | This study |
| pET28a-NmpU | Full length NmpU | This study |
| pET28a-NmpU^H96A^ | Full length NmpU^H96A^ | This study |
| pCD127 | *lacZ* expression | (3) |
| pCD127-*mxan_4236pro* | *lacZ* expression | This study |
| pCD127-*mxan_4236pro-*S1* | *lacZ* expression | This study |
| *pCD127-mxan_4236pro-*S2* | *lacZ* expression | This study |
| *pCD127-mxan_4236pro-*S1S2* | *lacZ* expression | This study |
| *pCD127-mxan_4236pro-σ^54^** | *lacZ* expression | This study |
| pCD127-*mxan_1578pro* | *lacZ* expression | This study |
| pCD127-*nmpUpro* | *lacZ* expression | This study |
| pCD127-*mxan_5543pro* | *lacZ* expression | This study |
| pCD127-*mxan_3966pro* | *lacZ* expression | This study |

^1^ In this table all references to “pro” indicate the promoter region for that gene. For example, pCD127-*mxan_3966pro* is the pCD127 plasmid backbone with the promoter for *mxan_3966* inserted upstream of the *lacZ*.

**References:**

1. Müller S, Willett JW, Bahr SM, Darnell CL, Hummels KR, Dong CK, Vlamakis HC, Kirby JR. 2013. Draft Genome Sequence of Myxococcus xanthus Wild-Type Strain DZ2, a Model Organism for Predation and Development. Genome Announc 1:e00217-13.
2. Bretl DJ, Müller S, Ladd KM, Atkinson SN, Kirby JR. 2016. Type IV-pili dependent motility is co-regulated by PilSR and PilS2R2 two-component systems via distinct pathways in Myxococcus xanthus. Mol Microbiol 102:37–53.
3. Bretl DJ, Ladd KM, Atkinson SN, Müller S, Kirby JR. 2018. Suppressor mutations reveal an NtrC-like response regulator, NmpR, for modulation of Type-IV Pili-dependent motility in Myxococcus xanthus. PLoS Genet 14:e1007714.

**Table S2.** Primers used in this study (5’ to 3’)

| **Primer** | **Sequence** | **Description** |
| --- | --- | --- |
| PilRProbe1fwd | CTCCTCGCAGTGAATTGGTGG | EMSA |
| ProbePil0aRev | AGTTGCGCAGAGAGTGCACC | EMSA, Probe A |
| ProbePil0bcRev | CGTGCCAGACGATCCGACTC | EMSA, Probe B |
| ProbePil0bFwd | GGTGCACTCTCTGCGCAACT | EMSA, Probe C, E |
| ProbePil9aRev | GTGGATCCACCCGGAGCA | EMSA, Probe C, D |
| ProbePil0cFwd | CATGCGCGGCTCGGC | EMSA, Probe D |
| ProbePil1Rev | TCACAGCGGCAACCCC | EMSA, Probe E |
| 4236proF | AAATCTAGACTGGAGTCGTAGACGTGCACC | EMSA |
| 4236proR | AAAGGATCCCATGGCGGTACTCCTGGCA | EMSA |
| Mxan0968probefwd | GCCGTCAGGTTGACATGG | EMSA |
| Mxan0968proberev | GGCGTGGCGCTCC | EMSA |
| Mxan1578probefwd | CAACGATTCGAAGGTGAGAGG | EMSA |
| Mxan1578proberev | GGGCGCGGCGTTCC | EMSA |
| Mxan3966probeFwd | GACAAAATCCTCCGTTCGTTGAGG | EMSA |
| Mxan3966probeRev | GTCTTCTTTTCTGGGCATGGCAC | EMSA |
| Mxan4246probeFwd | CGGCACACCCGTGTCAAG | EMSA |
| Mxan4246probeRev | GAGTCCTGGGGTAAGAGCCT | EMSA |
| Mxan5532probeFwd | CGGACTCGGCCTCC | EMSA |
| Mxan5532probeRev | CTCACCGATTCACCATTCCAAG | EMSA |
| Mxan5543probeFwd | CCAAGCGCGAACGCGG | EMSA |
| Mxan5543probeRev | TCACGATGTCACCTTGCGAAC | EMSA |
| Mxan6199probeFwd | GAGGGCGTCGCTCCTG | EMSA |
| Mxan6199probeRev | GCGAGCCGTTCCCGCTAC | EMSA |
| Mxan7061probeFwd | AGGCCCGACGCCCAAC | EMSA |
| Mxan7061probeRev | TCGTGACGTCCTTTGAGTCGAGG | EMSA |
| Mxan7069probeFwd | AGTAGTCGAAGTAGGGCACCTG | EMSA |
| Mxan7069probeRev | GCGTTCACGATTCCACACGTCC | EMSA |
| Mxan7373probeFwd | GGGTTCGCCGTAGGGG | EMSA |
| Mxan7373probeRev | ATGCCCGTTGACGCGTTG | EMSA |
| 4236proF-XbaI | AAATCTAGACTGGAGTCGTAGACGTGCACC | *lacZ* reporter |
| 4236proR-BamHI | AAAGGATCCCATGGCGGTACTCCTGGCA | *lacZ* reporter |
| Mxan1578proF-XbaI | AAATCTAGACAACGATTCGAAGGTGAGAGG | *lacZ* reporter |
| Mxan1578proR-HindIII | AAAAAGCTTGGGCGCGGCGTTCC | *lacZ* reporter |
| Mxan3966proF-XbaI | AAATCTAGAGACAAAATCCTCCGTTCGTTGAGG | *lacZ* reporter |
| Mxan3966proRHindIII | AAAAAGCTTGTCTTCTTTTCTGGGCATGGCAC | *lacZ* reporter |
| Mxan4246proF-XbaI | AAATCTAGACGGCACACCCGTGTCAAG | *lacZ* reporter |
| Mxan4246proRHindIII | AAAAAGCTTGAGTCCTGGGGTAAGAGCCT | *lacZ* reporter |
| Mxan5543proFXbaI | AAATCTAGACCAAGCGCGAACGCGG | *lacZ* reporter |
| Mxan5543proRHindIII | AAAAAGCTTTCACGATGTCACCTTGCGAAC | *lacZ* reporter |
| PilRpro2NmpRchangeF | ACTAAAAAACCCGCTGGCGTCCATGCG | Mutagenesis, S2 |
| PilRpro2NmpRchangeR | TCGTTTTTCAGTTGCGCAGAGAGTGCACCTC | Mutagenesis, S2 |
| PilRprosigma54F | GACGAAAGGGTCGAAGTCACCGCGCC | Mutagenesis, σ^54^ |
| PilRprosigma54R | ACGTGTTTGTGGATCCACCCGGAGCATG | Mutagenesis, σ^54^ |
| 4236pro5changefwd | CTAAAAAGCGACGGGCCTCCGCGCAA | Mutagenesis, S1 |
| 4236pro5changerev-2 | GCATTTTTTGGTCGACAAGCGCTTGCGC | Mutagenesis, S1 |
| 4236prosite2chgF | CTTAAAAACCCCAAGGCAGGGAAACA | Mutagenesis, S2 |
| 4236prosite2chgR | CTTTTTTTGGAGGCCCGTCGCTTTTT | Mutagenesis, S2 |
| 4236proquicksigma54-F | GAGAAAAAACGATGAAGGGACTATCGTCGTC | Mutagenesis, σ^54^ |
| 4236proquicksigma54-R | TGGGGTTTACAGGTCTGCCCCAGGGA | Mutagenesis, σ^54^ |
| NmpRD54A-Fwd | GGTGCTCACCGCCGTGCGCATGC | Mutagenesis |
| NmpRD54E-F | GGTGCTCACCGAAGTGCGCATGCC | Mutagenesis |
| NmpRquickDE-R | ACCGCGGGGGAGAACTCG | Mutagenesis |
| NmpRV87E-Fwd | GTTCGCCAGCGAGGAGACGGCCG | Mutagenesis |
| NmpRV87E-Rev | GCCGTCATCATCACGAAGGTGGC | Mutagenesis |
| NmpUH96AFor | TGGCCGCATGGCCGTGCGCATCGCCC | Mutagenesis |
| NmpUH96ARev | ATGCGGCAGCGCAGCGCG | Mutagenesis |
| 4246FullFwdNdeI | AAACATATGGCGGAAACCTTGTTCG | Protein Production |
| 4246HKRevHindIII | AAAAAGCTTTCATCCAACAGGAGGTACTGGC | Protein Production |
